# Semantic Associations Dominate Over Perceptual Associations in Vowel–Size Iconicity

**DOI:** 10.1177/2041669519861981

**Published:** 2019-07-12

**Authors:** Hideyuki Hoshi, Nahyun Kwon, Kimi Akita, Jan Auracher

**Affiliations:** Department of Language and Literature, Max Planck Institute for Empirical Aesthetics, Frankfurt, Germany; Department of English Linguistics, Graduate School of Humanities, Nagoya University, Japan; Department of Language and Literature, Max Planck Institute for Empirical Aesthetics, Frankfurt, Germany

**Keywords:** sound iconicity, perceptual processing, conceptual processing, frequency code, size-pitch association, Implicit Association Test

## Abstract

We tested the influence of perceptual features on semantic associations between the acoustic characteristics of vowels and the notion of size. To this end, we designed an experiment in which we manipulated size on two dissociable levels: the physical size of the pictures presented during the experiment (perceptual level) and the implied size of the objects depicted in the pictures (semantic level). Participants performed an Implicit Association Test in which the pictures of small objects were larger than those of large objects – that is, the actual size ratio on the semantic level was inverted on the perceptual level. Our results suggest that participants matched visual and acoustic stimuli in accordance with the content of the pictures (i.e., the inferred size of the depicted object), whereas directly perceivable features (i.e., the physical size of the picture) had only a marginal influence on participants’ performance. Moreover, as the experiment has been conducted at two different sites (Japan and Germany), the results also suggest that the participants’ cultural background or mother tongue had only a negligible influence on the effect. Our results, therefore, support the assumption that associations across sensory modalities can be motivated by the semantic interpretation of presemantic stimuli.

## Introduction

### Sound Iconicity of Magnitude

While the relation between the form of a linguistic sign and its meaning is generally believed to be based on social conventions and thus to be arbitrary, there is also solid evidence suggesting that the articulatory and acoustic properties of phonemes in human language are implicitly associated with nonacoustic characteristics such as shape, size, and taste (for reviews, see Cuskley & Kirby, 2013; Hinton, Nichols, & Ohala, 1994; Nuckolls, 1999; Perniss, Thompson, & Vigliocco, 2010; Perniss & Vigliocco, 2014; Reay, 1994; Schmidtke, Conrad, & Jacobs, 2014; Svantesson, 2017). Research on phonosemantic relations has long been dominated by the attempt to prove its existence (Lockwood & Dingemanse, 2015), and only recently have approaches begun to distinguish between different kinds of sound–meaning relations (Sidhu & Pexman, 2018). Generally, associations between a language’s sound and meaning are referred to as *sound symbolism*. However, as in the Peircean tradition of semiotics, the term *symbolism* is reserved for signs whose relation to their meaning is arbitrarily imputed (Peirce, 1867, p. 294); we prefer the term *sound iconicity* – that is, a relation between sound and meaning based on intrinsic resemblance (Wescott, 1971). In addition, we also wish to distinguish sound iconicity from onomatopoeia – that is, sound–meaning relations based on imitation. We thus use the term *sound iconicity* to refer to a perceived relation between the acoustic characteristics of a phoneme and nonacoustic features of sensory input that exist independently of a specific culture or language. Comparable definitions of sound iconicity have also been introduced elsewhere (e.g., Dingemanse, Blasi, Lupyan, Christiansen, & Monaghan, 2015; Hinton et al., 1994); however, due to differences in research interests, the terminology has yet to be used consistently.

Previous studies have provided solid evidence suggesting that sound iconicity plays an important role in natural language processing. For one, several studies have found a systematic relation between the relative occurrence of certain phonemes in a text and the text’s emotional tone (e.g., Auracher, Albers, Zhai, Gareeva, & Stavniychuk, 2011; Fónagy, 1961; Hevner, 1937; Whissell, 1999, 2000, 2004; but see Kraxenberger & Menninghaus, 2016; Miall, 2001). Other experimental studies have reported that participants can match words in their own language with those in an unknown foreign language more accurately than at a chance level, which indicates the existence of language-independent sound–meaning associations (Brackbill & Little, 1957; Brown, Black, & Horowitz, 1955; Brown & Nuttall, 1959; Gebels, 1969; Kunihara, 1971). There is also evidence suggesting that sound iconicity can support the acquisition of vocabulary in both foreign-language learning and early first-language acquisition (Imai & Kita, 2014; Imai, Kita, Nagumo, & Okada, 2008; Kantartzis, Imai, & Kita, 2011; Lockwood, Dingemanse, & Hagoort, 2016; Monaghan, Shillcock, Christiansen, & Kirby, 2014; Nygaard, Cook, & Namy, 2009; Perry, Perlman, & Lupyan, 2015). Studies that have compared the relation between the lexical meaning and the phonetic characteristics of words across languages have even provided solid evidence that there are some sound–meaning mappings commonly found throughout the world’s languages (Blasi, Wichmann, Hammarstrom, Stadler, & Christiansen, 2016; Ultan, 1978).

A well-studied example is the *sound iconicity of magnitude*, according to which there is a near-universal tendency to associate front vowels – such as/i/or/e/– with smallness, whereas back vowels – such as/o/and/u/– are more readily related to largeness (Becker & Fisher, 1988; Birch & Erickson, 1958; Greenberg & Jenkins, 1966; Miron, 1961; Newman, 1933; Oyama & Haga, 1963; Peña, Mehler, & Nespor, 2011; Preziosi & Coane, 2017; Sapir, 1929; Shinohara & Kawahara, 2016; Shrum, Lowrey, Luna, Lerman, & Liu, 2012; Thompson & Estes, 2011). The characterisation of vowels as *front* versus *back* refers to the relative position of the tongue (towards the front or back of the vocal tract) during their pronunciation. As the opposition between the articulatory characteristics of front vowels versus back vowels influences the frequency of the second formant (Ladefoged & Disner, 2012), the sound iconicity of magnitude can be explained by the *Frequency Code*, according to which low-frequency sounds are preferably associated with largeness, strength, and generally physical or social dominance, whereas the opposite holds for high-frequency sounds (Ohala, 1994; Ohtake & Haryu, 2013; Tsur, 2006; Walker & Smith, 1984).

### Sound Iconicity Is a Specific Form of Cross-Modal Associations

Sound iconicity can be defined as a specific form of *cross-modal* or *synaesthetic association* – that is, the association between stimuli of different sensory modalities due to an actual (or perceived) relation between seemingly unrelated characteristics of these stimuli (Spence, 2011). Such cross-modal associations have been found, for example, between brightness and pitch (Marks, 1974), pitch and spatial frequency (Evans & Treisman, 2010), or taste and pitch (Crisinel & Spence, 2009). According to Parise and Spence (2013), attempts to explain cross-modal associations can generally be subdivided into three different approaches: *structural correspondence*, which refers to equivalences in the neural processing of certain amodal attributes of sensory input; *statistical correspondence*, which is based on a frequent co-occurrence of certain stimuli attributes in nature; and *semantic correspondence*, which is a correspondence between perceivable features due to a shared association with abstract semantic concepts.

The distinction between different forms of cross-modal correspondence (i.e., structural vs. statistical vs. semantic correspondence) clearly does not imply that these forms are mutually exclusive. For example, it has been suggested that the statistical correspondence between body mass and the frequency of acoustic resonance is used in human- and nonhuman interaction to signal not only physical attributes but also social relations (Ohala, 1984). As size is usually directly related to strength and strength often determines an individual’s access to resources in most nonhuman species, a common strategy is to try to appear larger by erecting hairs or feathers to intimidate opponents. According to Morton (1977), some species use voice pitch in a similar fashion. By taking advantage of the association between sound frequency and body mass, animals of various species apply specific characteristics of their voice to convey the impression of size and strength. In line with this theory, sound frequency (of the fundamental or the formants) has been found to correlate with access to mating partners, to settle territorial fights among conspecifics, and to signal social dominance (e.g., Bowling et al., 2017; Charlton, Ellis, Brumm, Nilsson, & Fitch, 2012; Charlton & Reby, 2016; Colleye & Parmentier, 2012; Faragó et al., 2010; Vannoni & McElligott, 2008). On the other hand, individuals who try to appease a potentially aggressive dominant opponent aim to appear as harmless as possible and thus produce sounds with a relatively high pitch (Morton, 1994). Interestingly, studies on humans have repeatedly found that the assessment of a person’s social status by others is heavily influenced by acoustic characteristics related to the sound frequency in this person’s voice (Borkowska & Pawlowski, 2011; Klofstad, Anderson, & Nowicki, 2015; Klofstad, Anderson, & Peters, 2012; Ponsot, Burred, Belin, & Aucouturier, 2018; Puts, Gaulin, & Verdolini, 2006; Puts, Hodges, Cárdenas, & Gaulin, 2007). These findings are remarkable insofar as size and strength are not the main factors that determine social hierarchies in most human societies. Leongómez, Mileva, Little, and Roberts (2017) even reported that participants in job interviews adjust their vocal parameters (i.e., the frequency of the fundamental) to the perceived social status of their communication partner in relation to their own social status. It thus appears that over the course of evolution, the social relevance of the natural relation between size and pitch initiated a development towards a semantic association between sound frequency and an abstract concept of dominance (cf. Ohala, 1984; Watkins & Pisanski, 2016).

### Objectives, Hypothesis, and Implementation of the Study

Cross-modal associations could, thus, occur either on the level of directly perceivable features of stimuli (perceptual level) or on a higher level of cognitive processing that involves a semantic interpretation of a stimulus (conceptual level). Consider, for example, a drawing of a lion. On the perceptual level, the drawing uses certain colours and shapes, whereas on the semantic level, these colours and shapes are interpreted as a representation of an animal with certain attributes, such as strength, vitality, and so forth. Asked to assess the relation between the picture and an arbitrary sound, participants could either focus on correspondences between directly perceivable features, for example, whether the hue of the colour and the acoustic frequency of the sound match, or on correspondences between the sound and the content of the picture, for example, whether the pitch of the sound reflects the majestic aura of the depicted animal. At least for synaesthetes, studies suggest that cross-modal associations mostly rely on the semantic interpretation of a stimulus rather than on its directly perceivable appearance (Nikolić, 2009). Nikolić, therefore, suggested to use the term *ideasthesia* instead of synaesthesia to emphasize that cross-modal associations should be conceptualised as a specific type of semantic associations, whereby semantic concepts are wired to sensory activation.

Evidence according to which the same might also apply for nonsynaesthetes has recently been reported by a study that focused on the specific role of perceptual versus conceptual processing in sound iconicity (Auracher, 2017). Results of this study suggest that cross-modal associations between pseudowords and pictures were dominated by the content of the pictures, whereas physical properties – namely the size of the pictures – had no measurable influence. These findings contrast those of previous studies. For example, Parise and Spence (2012), who used a similar research design found evidence for implicit cross-modal associations between nonsemantic (i.e., meaningless) stimuli, such as grey discs or pure sine-wave tones. As these stimuli had no semantic level (i.e., no content), the measured effect can be attributed only to associations based on directly perceivable features. Similarly, Gallace and Spence (2006) reported that the frequency of a task-irrelevant acoustic prime (pure sine-wave tone) influenced the performance of participants in a speeded recognition task. Similar to Parise and Spence (2012), Gallace and Spence (2006) used grey discs of different sizes as visual stimuli, suggesting that participants associated visual and acoustic stimuli due to perceivable features. At the same time, Gallace and Spence also reported a similar effect when using semantic stimuli (i.e., words). The authors, thus, conclude that synesthetic associations ‘are based on a presemantic/semantic processing at a stage in which an abstract, amodal representation of the stimuli’ might be set up (p. 1201). In an earlier study, Walker and Smith (1984) tested cross-modal associations between acoustic pitch and words referring to various sensory or emotional qualities using a Stroop interference design. Consistent with the results obtained by Gallace and Spence, Walker and Smith found evidence suggesting that directly perceivable features of stimuli, such as brightness, also contribute to cross-modal associations across sensory modalities due to suprasensory qualities, for example, the brightness of sounds. However, while there is evidence that cross-modal associations between size and pitch can be elicited by either directly perceptual or conceptual features of stimuli, to the best of our knowledge, no study has yet tested the interaction between these two levels of cognitive processing.

In the experiments presented here, we replicated the results reported by Auracher (2017) and also addressed two major shortcomings in the study. For one, in Auracher (2017), the influence of manipulations of perceptual features (i.e., physical size) was tested between, but not within, participants – that is, while the study revealed that manipulations of the perceptual features could not alter or eliminate the effect of semantic associations, the design did not allow for monitoring interactions between perceptual and semantic features. Consequently, it is possible that subtle yet significant influences of perceptual features on semantic associations remained undetected. Moreover, the experiments reported in Auracher (2017) were conducted solely in Japan, meaning that the study design did not allow for testing the influence of participants’ linguistic and cultural backgrounds.

In line with Auracher (2017), the current study was based on the assumption that size can be conceptualised on two different levels: on a perceptual level and a semantic level. Throughout this article, we use the term *perceptual level* to refer to directly perceivable features, such as the physical size of a picture, whereas the term *semantic level* is used to refer to properties related to the content of a picture, such as the inferred real-world size of a depicted object. As the study design allowed us to manipulate the perceptual level and the semantic level of size independently of each other, we were able to test the relative influence of perceptual and semantic features on cross-modal associations.

To this end, we applied an *Implicit Association Test* (IAT; Greenwald, McGhee, & Schwartz, 1998). In the IAT, participants perform a speeded categorisation task under two different conditions: one Congruent and one Incongruent condition (see Material and Methods section for details). In the Congruent condition, presumably associated visual and acoustic stimuli are allocated to the same response behaviour. In contrast, in the Incongruent condition, presumably associated stimuli are allocated to opposed response behaviours. The test predicts that participants should have fewer problems and thus be able to complete the task more quickly and with fewer mistakes in the Congruent condition compared with the Incongruent condition. In the following paragraphs, we refer to differences in response time (RT) between the conditions with the abbreviation ΔRT, with a positive ΔRT indicating that participants performed the task faster in the Congruent condition than in the Incongruent condition.

Based on the results reported by Auracher (2017), we hypothesised that cross-modal associations between visual and acoustic stimuli will be dominated by the semantic features of the visual stimuli, that is, the content of the pictures. Thus, our hypothesis is corroborated if participants perform the task better, that is, faster and with less mistakes, in the conforming condition when compared with the nonconforming condition. On the other hand, there is evidence suggesting that directly perceivable features of visual and acoustic stimuli can also have an influence of participants’ performance in speeded recognition tasks (e.g., Gallace & Spence, 2006; Parise & Spence, 2012; Walker & Smith, 1984). The aim of the current study, thus, is to monitor to what extent perceivable features of stimuli have a measurable effect on semantic-based cross-modal associations.

**Figure 1.**
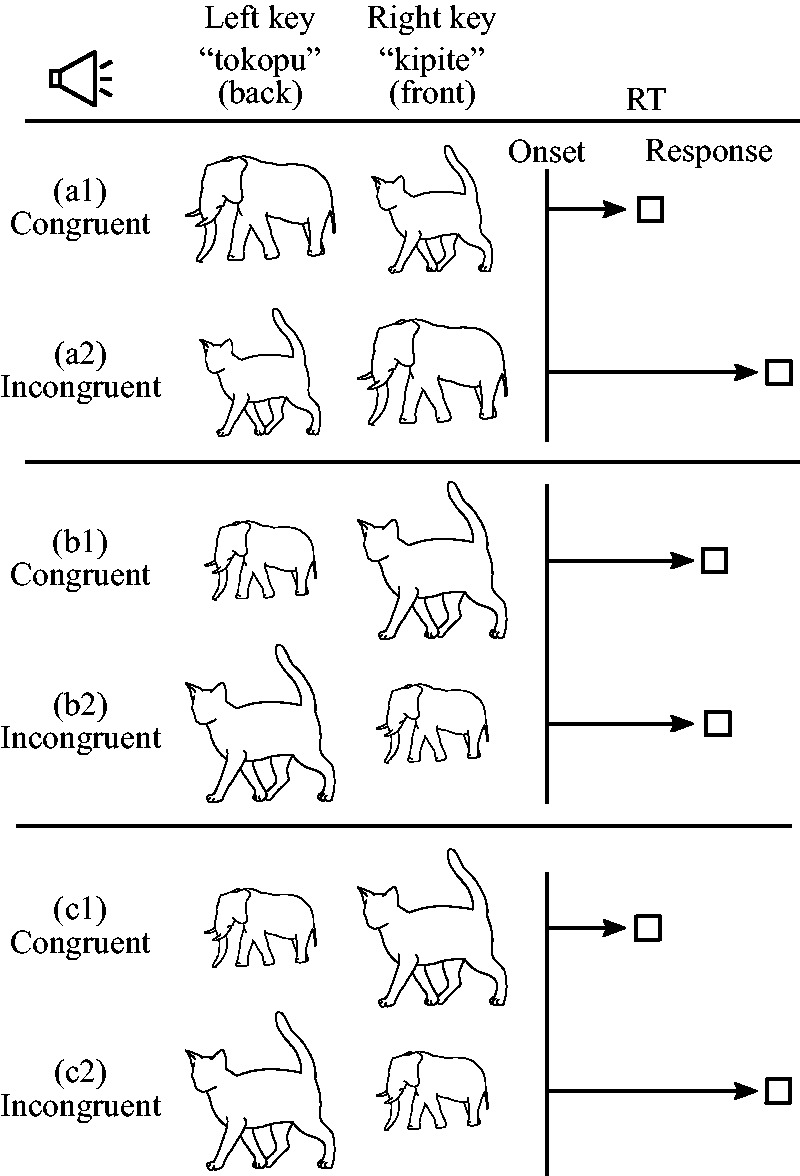
Schematic description of the hypothesis and expected results. (a) Original condition: Cross-modal associations between low-frequency sounds (‘tokopu’) and largeness (elephant) or between high-frequency sounds (‘kipite’) and smallness (cat) lead to a shorter response delay (a1) than the opposite combination (a2). (b) Counter hypothesis: Perceptual features interfere with semantic cross-modal associations, thus significantly reducing the differences in the response delay between (b1) and (b2) if the physical size of the picture and the actual size of the depicted animal are in opposition. (c) Null hypothesis: Perceptual features are suppressed during semantic cross-modal associations. Consequently, incongruity between the physical size of the picture and the actual size of the depicted animal has no influence on participants’ performance. RT = response time.

We tested the hypothesis by experimentally modifying the physical size of the visual stimuli in the IAT paradigm. In the present study, modifications of physical size always resulted in an opposition between perceptual features and semantic features (i.e., large objects were depicted with a smaller image than were small objects, and vice versa). Thus, if perceptual features have a measurable influence on cross-modal semantic associations, the manipulation of the physical size of the pictures should always lead to a reduction or even inversion (from plus to minus) of the ΔRT. In contrast, finding that the ΔRT remains unaffected by the manipulation of the visual stimuli would clearly suggest that any effects of perceivable features are ignored or suppressed when processing cross-modal associations that are based on semantic features (Figure 1).

Moreover, as the manipulation of the physical size of the visual stimuli was altered over multiple gradations, it was possible to monitor the effect of perceptual features on semantic associations in relation to the degree of the manipulation. It is thus possible to speculate that the degree of influence of perceptual features on semantic associations increases steadily with the degree of opposition between perceptual and semantic features. Alternatively, it could be assumed that there is a certain threshold at which the attention of the participants shifts from semantic features to perceptual features.

Finally, we conducted the experiment with participants from two different linguistic and cultural backgrounds – namely Japan and Germany – which allowed us to control potential interference from sound congruency between the names of the objects depicted in the visual stimuli and the phonetic characteristics of the acoustic stimuli. In other words, as the objects depicted in the visual stimuli had entirely different names with different phonetic characteristics in the two languages investigated, possible congruencies between the name of an object and the acoustic stimuli in one language were ineffective in the other language. Consequently, effects that were found independently of participants’ cultural background cannot be attributed to cultural or language-specific causes.

## Material and Methods

### Participants

The experiment was performed at two different sites: Nagoya University in Aichi, Japan, and the Max Planck Institute for Empirical Aesthetics in Frankfurt, Germany. At Nagoya University, 35 native Japanese speakers (20 female, 3 left-handed, mean age 23.4 ± 5.2 years) took part in the study, whereas at the Max Planck Institute, 34 native German speakers (19 female, 4 left-handed, mean age 24.4 ± 3.6 years) took part. Among the participants, one Japanese speaker and seven German speakers reported that they had additional cultural backgrounds, and two Japanese speakers and five German speakers were bilingual. All participants reported having no hearing impairments and had normal or corrected-to-normal vision. All experimental procedures (e.g., verbal instructions, screen messages) were carried out in the speakers’ native language in the respective country. All experimental procedures were ethically approved by the Ethics Council of the Max Planck Society and were undertaken with the written informed consent of each participant.

### Stimuli

**Figure 2.**
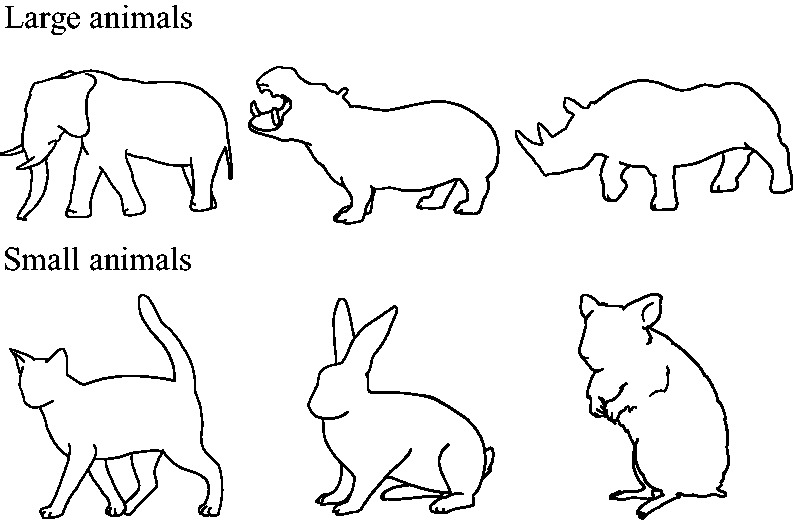
Pictures of large and small animals used as visual stimuli.

The stimuli used in the experiment were taken from Auracher (2017). The visual stimuli consisted of six line drawings depicting either large or small animals (Figure 2). The pictures were drawn by a professional illustrator (NiKo Illustration; http://www.niko-illustration.de). The illustrator was instructed to draw either ‘big, strong, and heavy animals’ or ‘small, weak, and light animals’. Otherwise, the illustrator was uninformed with regard to the goal of the research. All animals were illustrated with black lines and in a three-quarter left profile. For the purposes of this study, the colour of the stimuli was digitally edited to make the line drawings white against a transparent background. The original scale of each animal was adjusted to minimise differences between the pictures regarding the area (number of screen pixels) that was occupied by the body of each animal (see Supplementary Table S1 for the details).

To avoid an inflation of the conditions, we selected three animals per category from the original set of stimuli, which contained eight animals per category. The selection of the visual stimuli guaranteed that participants would recognise the depicted animals and their categories (i.e., small or large) easily and coherently. According to the pretest conducted by Auracher (2017), participants did not report major problems in recognising the animals, and they consistently categorised the large animals into the large category and small animals into the small category. In addition, the phonetic characteristics of the names of the animals did not favour associations between visual and acoustic stimuli in the predicted direction. Regarding the phonetic characteristics, we carefully controlled the distribution of front and back vowels in animal names across categories (small vs. large) and languages such that the total occurrence of front vowels in the names of the large animals was equal to or higher than the total occurrence of back vowels, whereas the relation was inverse for small animals (Supplementary Table S1). In other words, large animals had a ratio of front to back vowels of 4:1 in German and 1:1 in Japanese, while small animals had a ratio of 0:0 in German and 2:3 in Japanese. Some of the animals were in clear opposition regarding their phonetic characteristics when comparing the Japanese and German names. For example, the German word for elephant (*Elefant*) contains two front vowels but no back vowel, whereas the Japanese word for elephant (*Zou*) contains only back vowels but no front vowels. The opposite is true for the rhinoceros, which contains one back vowel but no front vowels in German (*Nashorn*) but one front vowel and no back vowels in Japanese (*Sai*; see Supplementary Table S1 for a phonetic transcription of each item). Thus, if the congruence between phonetic characteristic of the animal name and its size influenced the results, Japanese participants would have had fewer problems in matching the elephant with pseudowords containing back vowels, while the rhinoceros would have been perceived as being closer to pseudowords containing front vowels. At the same time, the inverse relation should have been found for German participants.

For the acoustic stimuli, we used three pseudowords for each category, which were also used in Auracher (2017). These pseudowords were generated by creating sequences of three syllables, each consisting of one consonant and one vowel (CVCVCV). As Japanese, in contrast to German, has no consonant clusters, the structure of the pseudowords was closer to typical Japanese words compared with typical German words. However, lexical items with regular consonant–vowel intervals can also be found in German and are, thus, not unusual for native German speakers. Moreover, the main research question, that is, the association between visual and acoustic stimuli, was not affected by the phonetic differences between German and Japanese.

The two categories of pseudowords differed with respect to their vowels: While the first one contained back vowels (/o/and/u/), the other contained front vowels (/i/and/e/). To avoid an influence of the consonants, the plosive consonants/p/,/t/, and/k/were used for both categories of pseudowords. The distribution of the consonants across the three syllables followed the same pattern in both categories (e.g., both ‘tokopu’ and ‘tikipi’ had the same sequence of consonants). Three pseudowords per category were recorded by a male speaker (pseudowords with back vowels: tokopu, kopotu, pokotu; pseudowords with front vowels: tikipi, kipite, pikite). The recordings were created in an anechoic room using a Roland CD-2e digital recorder. The speaker was asked to clearly put the stress on the first syllable when pronouncing each word. Otherwise, he was uninformed with respect to the aim of the experiment. The length of the recordings was manipulated to fit exactly 0.58 s, and the volumes were normalised between the stimuli. The files were saved in WAV 16-Bit PCM format.

### Apparatus

At the Japanese site, we used a MacBook Air (MacBook Air 6.1; Mac OS 10.10.1) to display the stimuli. The computer was connected to a 24-inch screen (LCD-AD202X: I-O DATA, Kanazawa, Japan) with a resolution of 1,600 × 900 pixels at 60 Hz. Acoustic stimuli were presented using headphones (MDR-NC500D: SONY, Tokyo, Japan). At the German site, we used a Windows PC and a 24-inch screen (XL2420Z: BenQ, Taipei, Taiwan) with a resolution of 1,920 × 1,080 pixels at 60 Hz. Acoustic stimuli were presented using headphones (DT 770 PRO: BEYERDYNAMIC, Heilbronn, Germany). At both sites, experimental procedures were controlled via Psychtoolbox 3 (www.psychtoolbox.org), which ran in MATLAB (Version 9.2.0; MathWorks, Na-tick, MA, USA). The response keys (the ‘C’ and ‘M’ keys) on the keyboard were marked with nontransparent stickers, which suppressed the influence of the characters. The participants were seated about 57 cm away from the monitor and instructed to minimise their motion during the task (and to keep their distance to the monitor constant).

### The IAT

The design of the experiments conformed to the IAT (Greenwald et al., 1998). The IAT was designed to detect associations between stimuli of two different modalities. To this end, two sets of stimuli of two different modalities (visual and auditory) were each separated into two opposing categories (visual stimuli into large vs. small animals and auditory stimuli into pseudowords containing front vowels vs. pseudowords containing back vowels). Thus, the stimuli were separated into four categories (i.e., visual large, visual small, acoustic front vowel, and acoustic back vowel), two of which were assumed to be cross-modally associated (i.e., visual large with acoustic back vowel and visual small with acoustic front vowel). The design of the IAT conformed to a speeded categorisation test in which stimuli had to be sorted into one of two categories by pressing one of two response keys using either the left or the right index finger. During experimental blocks, stimuli from the two modalities were presented one at a time in a randomised order. Participants performed the test in two conditions: One Congruent condition in which the presumably cross-modally associated stimuli were allocated to the same response behaviour (e.g., large animals and pseudowords containing back vowels were allocated to the left button), and one Incongruent condition in which the presumably cross-modally associated stimuli were allocated to opposing response behaviours (e.g., large animals were allocated to the left button, and pseudowords containing back vowels were allocated to the right button). The test was based on the idea that participants perform better (i.e., faster and with fewer mistakes) in the Congruent condition than in the Incongruent condition. The order of the experimental blocks (first Congruent and second Incongruent, or vice versa) as well as the allocation of the categories to the left or right side during the Congruent block (e.g., small animals and pseudowords containing front vowels allocated to the left) was counterbalanced between participants.

### Blocks and Trials

**Figure 3.**
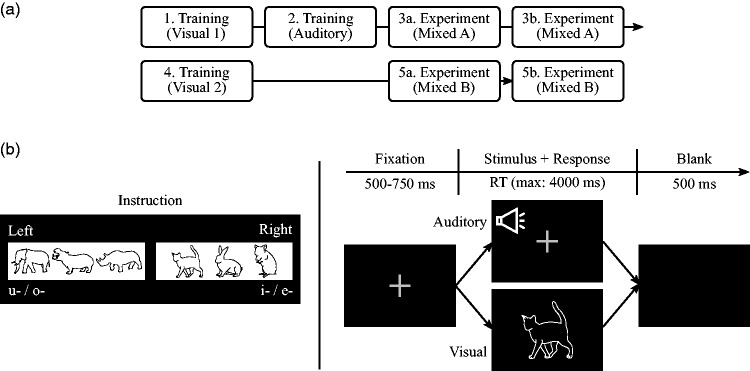
(a) Schematic representation of block structure in the experiment. Each participant completed five blocks consisting of three training blocks (Blocks 1, 2, and 4) and two experimental blocks (Blocks 3 and 5). Each experimental block was subdivided into two subblocks (i.e., 3a + 3b and 5a + 5b). In the training blocks, either visual or auditory stimuli were presented. In contrast, the two sensory modalities were mixed in the experimental blocks. The auditory category was always assigned to the same side throughout all blocks (e.g., front vowel to the left, back vowel to the right) and was trained in Block 2. The assignment of the visual category trained in Block 1 (Visual 1; e.g., small animals to the left, large animals to the right) was used in experimental Block 3. The side was then switched in training Block 4 (Visual 2; e.g., large animals to the left, small animals to the right), which was used in the subsequent experimental Block 5. Consequently, when combined with the auditory categories, experimental Blocks 3 and 5 fell into different conditions (e.g., Mixed A: Congruent condition; Mixed B: Incongruent condition). (b) Sample screens presented in the experiment. Note that the instruction screen has been simplified and translated from the original version for better visibility and comprehensibility.

Following the standard procedure of the IAT (Nosek, Greenwald, & Banaji, 2005), each experiment consisted of five blocks in total: three for training and two for the experiment (see Figure 3(a)). In the training blocks (Blocks 1, 2, and 4), participants practiced the allocation of the stimuli category for each modality separately (e.g., first for the visual modality and second for the auditory modality). Each experimental block was subdivided into two subblocks (i.e., Blocks 3a + 3b and 5a + 5b) to avoid an influence of fatigue on the task performance. In other words, the subblocks of each experimental block did not differ regarding the allocation of the categories to the left or right side. Training Block 4 was used to switch the allocation of the visual stimuli. Consequently, the experimental conditions changed between Blocks 3 and 5, with either Block 3 corresponding to the Congruent condition and Block 5 to the Incongruent condition, or vice versa.

Each stimulus was presented 6 times during the training blocks and 12 times during the experimental blocks, resulting in 36 trials for training blocks and 144 trials for experimental blocks (72 trials for each of the visual and auditory presentations). In two thirds of the visual trials (24 trials for training blocks and 48 trials for experimental blocks), visual stimuli were presented in the original size (100%; cat: 13.1° × 14.2°, rabbit: 11.5° × 13.8°, hamster: 9.3° × 14.4°, elephant: 14.2° × 9.5°, hippopotamus: 15.3° × 9.9°, rhinoceros: 16.9° × 8.3° in visual angle (VA)). In the remaining trials (12 trials for training and 24 trials for experimental blocks), the size of the pictures was manipulated such that large animals appeared smaller and small animals appeared larger. The experimental manipulation of size increased over four steps: ±16.25%, ±32.50%, ±48.75%, and ±65.00%. Manipulations beyond ±65% of the original size turned out to be either too small to be recognised or too large to fit into the display and were therefore not used in the study. Consequently, in the training blocks, each stimulus (visual or acoustic) was presented six times. For visual stimuli, pictures were presented in three different sizes – that is, four times in the original size and two times in a modified size (pseudorandomly taken from 100 ± 16.25%, 100 ± 32.50%, 100 ± 48.75%, or 100 ± 65.00%). In the experimental blocks, each of the 6 acoustic stimuli was presented 12 times, and each of the 6 visual stimuli was presented 12 times in 5 different sizes (100% × 8, 100 ± 16.25% × 1, 100 ± 32.50% × 1, 100 ± 48.75% × 1, and 100 ± 65.00% × 1).

The order of the trials was carefully controlled (see Supplementary Figure S1) to meet the following three criteria: (a) There could never be more than three trials with stimuli of the same category (e.g., large animals, front-vowel words, etc.) aligned in direct sequence, (b) there always had to be at least two baseline-sized trials (original 100% scale) inserted between trials of visual stimuli presented in nonoriginal size (±16.25, ±32.50, ±48.75, and ±65.00%), and (c) in the experimental blocks, there could never be more than two trials directly adjacent to each other in which stimuli that belonged to the same modality (visual or auditory) were presented. By inserting at least two baseline trials between trials with manipulated size (the second criterion), we aimed to establish the original size as baseline so that participants would perceive visual stimuli with manipulated size as deviating from the original. As a consequence, we expected to maximise the influence of the physical size changes in the visual trials.

### Procedure

Participants were seated in a dim room and instructed about the task. To ensure that the terminology did not prime participants’ cross-modal associations, we avoided using terms that directed the participants’ attention towards size (e.g., ‘large group’, ‘small group’) while giving instructions. Instead, we used deictic expressions (e.g., ‘this group’, ‘that group’, etc.) to verbally point to the categories while they were displayed on the screen. The acoustic groups were referred to by their phonetic characteristics; thus, we used the ‘i-/e- vowel group’ to refer to pseudowords containing front vowels and the ‘u-/o- vowel group’ to refer to pseudowords containing back vowels.

To confirm that participants had understood the instructions, they completed three sample trials before beginning the experiment. The instructor left the room after the sample trials, and the participants then began the experiment and proceeded through the blocks at their own pace. At the beginning of each block, the allocation of auditory or visual categories was presented, and the participants were instructed to memorise the allocation (Figure 3(b)).

In each trial, one stimulus – either visual or acoustic – was presented on the screen or via headphones. Participants were asked to categorise the presented stimulus as quickly and precisely as possible by pressing either the ‘C’ or the ‘M’ key (Figure 3(b)). Both answer keys were covered to ensure that the letters would not influence participants’ performance. All stimuli were preceded by a grey fixation cross (1.5° × 1.5° in VA) against a black background (Figure 3(b)). The presentation of the fixation cross lasted from between 500 and 750 ms. During the presentation of the acoustic stimuli, the fixation cross remained on the screen. If the categorisation was incorrect, a cross (X) was presented at the bottom of the screen, and participants were asked to correct the answer by pressing the other key.

### Data Processing

The data were analysed separately for Japanese and German participants. First, the average accuracy of the categorisation task (hit rate) was calculated for each block (Training, Congruent, and Incongruent blocks) and modality (visual and auditory). To test the effect of the condition and modality on accuracy, the dependent variable (average accuracy) was regressed using a liner mixed effect model (LMEM) with the fixed factors of Condition (three levels: Training, Congruent, and Incongruent) and Modality (two levels: visual and auditory) and the interaction term between the two. To take individual differences into account, a random intercept and random slopes (for all fixed predictors) were entered into the model for each participant. The model was estimated using the maximum likelihood method. Estimated fixed coefficients of fixed predictors were tested for the null hypothesis (with the coefficients being equal to zero) using a *t* test. Pairwise comparisons were conducted using Tukey-corrected tests. As the results showed that the error rate was extremely low for all conditions (<10%), we decided to exclude incorrect trials from the following analysis to reduce the danger of distorting the results by using one of the scoring algorithms to correct the response time (RT) of the error trials introduced by Greenwald, Nosek, and Banaji (2003).

We also examined the development of the RT over the course of each block to detect ‘training effects’. To this end, we plotted the RT for each condition and for each modality as a function of the trial number (the position of a trial within a block). Because each participant performed two independent training blocks in the visual modality (see Figure 3(a)), the RTs of the two blocks were averaged per trial number for the plot. For the other blocks, we used the original RT data. To test the ‘training effect’ separately per condition and modality, the training curve was fitted into a linear function, and the linear coefficient was estimated using a least squares algorithm (applying the ‘polyfit’ function in MATLAB). The estimated coefficients were then subjected to a one-sample *t* test and compared with zero. We next calculated the average RT for each block and for each modality. We tested the effect of the condition and modality on the RT by using LMEM with the fixed factors of Condition (three levels: Training, Congruent, and Incongruent) and Modality (two levels: visual and auditory). Because a significant ‘training effect’ was confirmed for Training and Incongruent conditions, the Trial Number (the position of a trial within a block) was used as a fixed covariate. The interaction terms between these three factors (but not the interaction of all three factors) were also used as fixed factors. Moreover, random intercept and slopes (for all fixed predictors) were entered into the model for each participant.

Next, we tested whether the category of the stimuli (e.g., large and small animals for the visual modality and back- and front-vowel words for the auditory modality) had an influence on the RT. To test the effect of visual and auditory modality separately, the RT was modelled by the LMEM with the two fixed factors of Condition (three levels: Training, Congruent, and Incongruent) and Category (two levels: large and small animals for visual, and front- and back vowels for auditory) and with one fixed covariate: Trial Number. Again, the random intercept and slopes (for all fixed predictors) were entered into the model for each participant. We further investigated the effect of stimulus Type (e.g., elephant vs. hippopotamus vs. rhinoceros) on the RT. In other words, we tested whether – for example – the RT significantly varied among the three large animals. To this end, the RT was modelled by the LMEM separately for each category (large animal, small animal, back-vowel words, and front-vowel words). Again, the LMEM comprised the two fixed factors of Condition (three levels: Training, Congruent, and Incongruent) and Type (three levels: three stimuli in the category) and one fixed covariate (Trial Number), and the random intercept and slopes (for all fixed predictors) were entered into the model for each participant. Finally, for the central purpose of the present study, we tested the effects on the RT related to manipulations of the physical size of the visual stimuli. In so doing, we focused on the visual trials in the experimental blocks alone. Again, we used the LMEM with RT as an independent variable, the fixed factor of Condition (two levels: Congruent and Incongruent), and the two fixed covariates of Size (100 ± 0%, 100 ± 16.25%, 100 ± 32.50%, 100 ± 48.75%, and 100 ± 65.00%) and Trial Number. Random intercept and slopes (for all fixed predictors) were entered into the model for each participant. For the Incongruent condition, the RT was significantly different between the subcategories of the visual stimuli (large and small animals); thus, the LMEM was built and tested separately per category (i.e., large vs. small animals). We further tested whether the difference in the RTs between Congruent and Incongruent conditions (ΔRT) was a function of the physical size of the stimuli in either a linear- or a quadratic fashion. We calculated the ΔRT by subtracting the average RTs for Congruent blocks from those for the Incongruent blocks, for each participant, for each category of the visual stimuli (large and small animals), and for each level of physical size of the stimuli (100 ± 0%, 100 ± 16.25%, 100 ± 32.50%, 100 ± 48.75%, and 100 ± 65.00%). To quantify changes of ΔRT as a function of physical size, the ΔRTs were fitted into one-dimensional (linear) and two-dimensional (quadratic) curves for each participant and for each subcategory using a least squares algorithm. We then tested whether the estimated linear- and quadratic trends were statistically meaningful by comparing each of the estimated coefficients (linear and quadratic) against zero using a one-sample *t* test.

All data analysis procedures and statistical tests were performed using MATLAB and its Statistics and Machine Learning Toolbox.

## Results

### Effects of Training and Experimental Conditions

**Figure 4.**
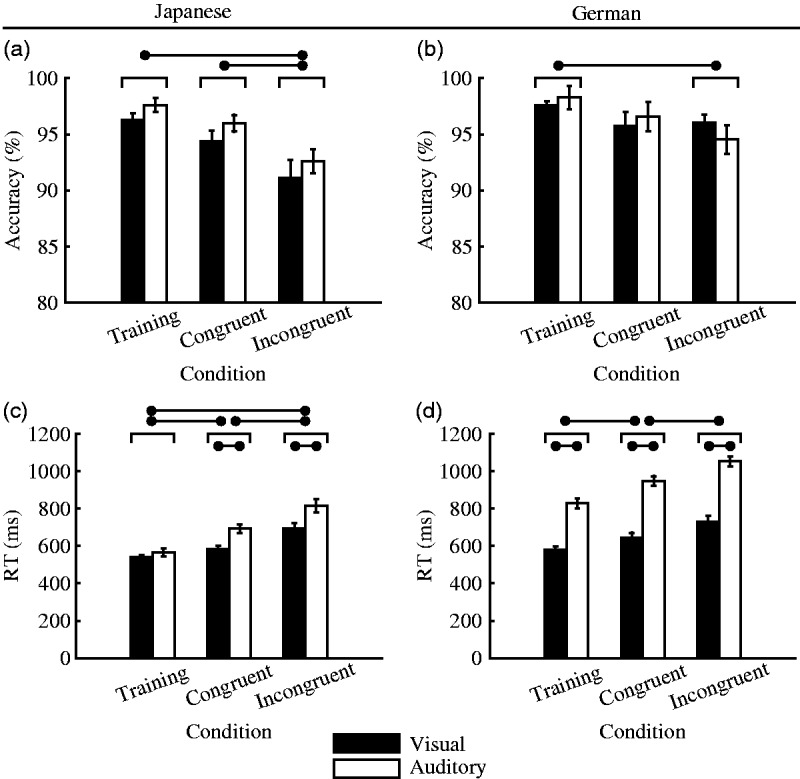
Average task performance accuracy (top figures: a and b) and RT (bottom figures: c and d) shown for each condition and modality. The figures were drawn using the dataset from Japanese (left figures: a and c) and German (right figures: b and d) participants. Error bars indicate standard errors. The results of pairwise comparisons are represented by horizontal bars (*p* < .05, Tukey-corrected). RT = response time.

The average task performance (accuracy and RT) for each condition, stimulus modality, and the nationality of the participants are shown in Figure 4. Regarding the accuracy data (Figure 4(a) and (b)), the results revealed a general trend for participants from both language groups to perform better in the training condition than in the experimental (Congruent and Incongruent) conditions. The result reflected the difficulty of the task; the training condition involved trials from a single modality (i.e., visual OR auditory), whereas the experimental conditions consisted of the trials from both modalities (i.e., visual AND auditory); thus, the latter was more difficult than the former. The comparatively good performance in the training condition indicated that participants trained the allocation of the stimuli categories (e.g., large animals to left, small animals to right) successfully.

For the Japanese data, this finding was supported by the LMEM (Table 1(a)), which showed that the fixed factor Condition = Training contributed significantly to the model, whereas it did not do so for the German data (*B* = 1.82, *p* = .131; Table 1(b)). Comparing the accuracy of the two experimental conditions, the Japanese data clearly showed that the Congruent condition had a higher accuracy than the Incongruent condition (Congruent condition = 95.32%, Incongruent condition = 92.02%; Figure 4(a) and *B* = –3.21, *p* = .029; Table 1(a)), but again, the German data did not show direct evidence (Congruent condition = 96.42%, Incongruent condition = 95.51%; Figure 4(b) and *B* = 0.31, *p* = .798; Table 1(b)). It is nevertheless important to note that the average accuracy was higher for the German data (> 95% for all conditions) compared with the Japanese data, which might account for the reported differences between Japanese and German accuracy data (see Discussion section).

**Table 1.** Results of the Liner Mixed Effect Model Analyses Performed on Accuracy (a and b) and the RT (c and d) Data: (a) Japanese Participants – Accuracy, (b) German Participants – Accuracy, (c) Japanese Participants – RT, and (d) German Participants – RT.

	*B*	*SE*	95% CI		*t*	*p*
(a) Accuracy: JP						
Intercept*	94.37	0.94	92.51	96.23	100.05	<.001
Condition = Training*	1.90	0.71	0.50	3.31	2.67	.008
Condition = Incongruent*	−3.21	1.46	−6.10	−0.33	−2.20	.029
Modality = Auditory*	1.63	0.58	0.49	2.77	2.82	.005
Training × Auditory	−0.28	0.84	−1.93	1.38	−0.33	.741
Incongruent × Auditory	−0.18	1.50	−3.13	2.77	−0.12	.905
(b) Accuracy: DE						
Intercept*	95.77	1.22	93.37	98.18	78.54	<.001
Condition = Training	1.82	1.20	−0.54	4.18	1.52	.131
Condition = Incongruent	0.31	1.20	−2.05	2.67	0.26	.798
Modality = Auditory*	0.80	0.34	0.13	1.47	2.35	.020
Training × Auditory	−0.10	1.07	−2.22	2.02	−0.10	.924
Incongruent × Auditory	−2.33	1.19	−4.68	0.02	−1.95	.052
(c) RT: JP						
Intercept*	595.19	22.97	550.17	640.21	25.91	<.001
Condition = Training	1.01	22.90	−43.88	45.89	0.04	.965
Condition = Incongruent*	149.42	22.92	104.49	194.35	6.52	<.001
Modality = Auditory*	106.03	16.90	72.91	139.15	6.28	<.001
Trial number	−0.17	0.14	−0.44	0.10	−1.24	.214
Training × Auditory*	−81.35	18.92	−118.44	−44.27	−4.30	<.001
Incongruent × Auditory	10.93	15.32	−19.10	40.96	0.71	.476
Training × Trial Number*	−2.88	0.55	−3.96	−1.80	−5.24	<.001
Incongruent × Trial Number*	−0.52	0.17	−0.86	−0.18	−3.03	.002
Auditory × Trial Number	0.06	0.14	−0.20	0.33	0.47	.638
(d) RT: DE						
Intercept*	661.16	32.31	597.83	724.49	20.46	<.001
Condition = Training*	−35.57	21.01	−76.74	5.61	−1.69	.090
Condition = Incongruent*	112.30	23.51	66.22	158.37	4.78	<.001
Modality = Auditory*	305.13	17.55	270.72	339.53	17.38	<.001
Trial number	−0.24	0.19	−0.61	0.13	−1.28	.201
Training × Auditory*	−57.19	25.84	−107.83	−6.54	−2.21	.027
Incongruent × Auditory	18.45	17.70	−16.24	53.14	1.04	.297
Training × Trial Number*	−2.23	0.48	−3.16	−1.30	−4.70	<.001
Incongruent × Trial Number	−0.37	0.20	−0.76	0.01	−1.90	.057
Auditory × Trial Number	−0.01	0.11	−0.23	0.21	−0.07	.941

*Note*. Congruent condition and visual modality are used as a reference level in the analysis. JP = Japan; DE = Germany; RT = response time; B = standardised beta coefficient of the predictor; SE = standard error; CI = confidence interval; *t* = *t* value; *p* = level of significance.

*p < .05.

In contrast, the RT data showed much clearer differences between the conditions, which were very close for Japanese and German participants. The results of pairwise comparisons revealed a clear trend for participants from both countries to have a shorter RT in the training condition than in the experimental conditions (Figure 4(c) and (d)). Again, the good performance of the participants in the training condition was used as an indicator that participants have understood the task and correctly learned the allocation of the stimuli. Regarding the effect of the experimental conditions, the RT was significantly shorter for the Congruent condition than for the Incongruent condition for both language groups (Japanese participants: Congruent condition: 638.22 ms, Incongruent condition: 757.51 ms; Figure 4(c) and German participants: Congruent condition: 794.45 ms, Incongruent condition: 885.28 ms; Figure 4(d)), which was also supported by the results of the LMEM (note that the fixed term Condition = Incongruent was significant for both models for Japanese and German RT data; Table 1(c) and (d)). This finding indicates that the performance was significantly worse in the Incongruent block than in the Congruent block, which corroborated our assumption that the pictures were implicitly associated with the phonetic characteristics of the vowels used in the pseudowords (semantic-level association) due to the implied size of the depicted animals. Thus, our results are in line with the assumption that participants implicitly associate large animals with pseudowords containing back vowels and small animals with pseudowords containing front vowels.

As a general trend (for both Japanese and German data), accuracy was higher for the acoustic stimuli than for the visual stimuli (average accuracy of Japanese participants: Visual = 94.03%, Auditory = 95.64%; German participants: Visual = 96.58%, Auditory = 96.59%), whereas the acoustic stimuli had a significantly longer RT than the visual stimuli (average RT of Japanese participants: Visual = 606.80 ms, Auditory = 694.66 ms; German participants: Visual = 647.37 ms, Auditory = 941.13 ms), indicating a speed-accuracy trade-off effect. Moreover, the difference of the average RTs between visual and acoustic stimuli was considerably greater for German participants than for Japanese participants, independently of the experimental condition (Figure 4(c) and (d)). Another interesting intercultural difference was that the German participants made relatively few mistakes but had a considerably longer RT than did the Japanese participants, which indicates that the German participants paid more attention to accuracy, while the Japanese participants placed a higher priority on speed. These differences notwithstanding, there was a noteworthy consistency between Japanese and German participants regarding the general effect of the experimental conditions and the stimulus modality on RT. For further analysis, we focused on the effect on RT alone as the data on accuracy suggested a strong ceiling effect (see Discussion section for details).

### Training Effect on RT

**Figure 5.**
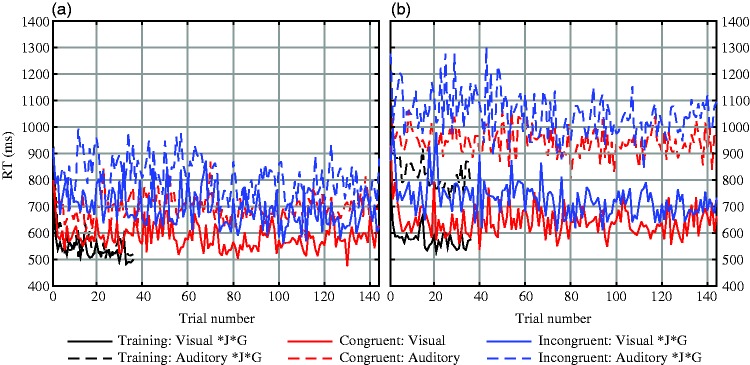
Average RT plotted along the trial number for each condition and modality for (a) Japanese and (b) German participants. *J: Estimated linear coefficients were significantly smaller than zero (*p* < .05) for Japanese data; *G: Estimated linear coefficients were significantly smaller than zero (*p* < .05) for German data. RT = response time.

Figure 5 displays the development of the RT over the blocks separately for each condition and modality. Although the intertrial differences are considerably high (i.e., trends are confounded by high-frequency noise), we still found that the average RT has a general tendency to decrease over the course of a block. Testing the slope of this trend line against zero by applying a one-sample *t* test (Table 2(a) and (b)) revealed that the performance of the participants from both countries significantly improved in the training condition and the Incongruent condition. By contrast, there was no significant improvement during the Congruent blocks. Our results thus suggest a training effect, meaning that participants’ task performance (reaction speed) improved while they were practicing the task. Moreover, the difference between the experimental conditions regarding this training effect implies that participants had fewer problems to automatically perform the task in the Congruent condition, whereas they had to train the implicit association between visual and acoustic stimuli while performing the task in the Incongruent condition.

**Table 2.** Results of One-Sample *t* Tests for Estimated Linear Coefficients of Training Curve Compared Against Zero.

	*M*	*SE*	95% CI		*t*	*p*
(a) JP						
Training: Visual*	−26.30	6.18	−38.86	−13.74	−4.25	<.001
Training: Auditory*	−42.84	7.46	−57.99	−27.68	−5.75	<.001
Congruent: Visual	−9.50	5.64	−20.97	1.97	−1.68	.101
Congruent: Auditory	−2.49	6.87	−16.45	11.47	−0.36	.719
Incongruent: Visual*	−27.32	7.23	−42.01	−12.63	−3.78	<.001
Incongruent: Auditory*	−27.82	9.87	−47.88	−7.76	−2.82	.008
(b) DE						
Training: Visual*	−20.61	4.28	−29.31	−11.91	−4.82	<.001
Training: Auditory*	−35.30	10.25	−56.16	−14.45	−3.44	.002
Congruent: Visual	−8.92	8.24	−25.68	7.84	−1.08	.287
Congruent: Auditory	−11.05	6.18	−23.62	1.53	−1.79	.083
Incongruent: Visual*	−27.56	9.01	−45.88	−9.24	−3.06	.004
Incongruent: Auditory*	−24.48	8.32	−41.42	−7.54	−2.94	.006

*Note*. JP = Japan; DE = Germany; M = mean; SE = standard error; CI = confidence interval; *t* = *t* value; *p* = level of significance.

*p < .05.

Again, these results were also supported by the LMEMs. For Japanese RT data, two interaction terms made a significant contribution (Training × Trial Number and Incongruent × Trial Number: Table 1(c)). For German RT data, the interaction term Training × Trial Number was a significant predictor in the model, while the interaction of Incongruent × Trial Number was marginally significant (Table 1(d)).

### Differences Between Categories and Individual Stimuli

Next, we tested whether the main effect of the experimental condition on RT was generalisable across all categories (e.g., large vs. small animals) and across all types of stimuli (e.g., elephant, hippopotamus, or rhinoceros). The data are visualised in Supplementary Figure S2 (Japanese data) and Figure S3 (German data), and the results of the LMEMs are shown in Supplementary Table S2 (Japanese data) and Table S3 (German data). Most important, we found that the RT was significantly longer in the Incongruent condition than in the Congruent condition for all categories and all types. The pairwise comparisons revealed significant differences between the two conditions for all categories/items (Supplementary Figures S2 and S3), and the LMEM revealed a significant contribution of the fixed term Condition = Incongruent in all models (Supplementary Tables S2 and S3). In addition, the results also confirmed that participants generally had shorter RT during the training blocks compared with the experimental blocks. The results of the pairwise comparisons thus always had the pattern of Training < Congruent < Incongruent (significant differences are represented by horizontal bars in Supplementary Figures S2 and S3).

The second important result is that we found no major differences regarding the RT between categories or individual stimuli. In most LMEMs, there were no significant contributions of the fixed predictors of Category or Type (e.g., ‘Category = Small’ in Supplementary Tables S2(a) and S3(a), ‘Type = Hippopotamus’ and ‘Type = Rhinoceros’ in Supplementary Tables S2(c) and S3(c)) or of the interaction terms of Condition × Category or Condition × Type (e.g., ‘Training × Front’ in Supplementary Tables S2(b) and S3(b) and ‘Training × kopotu’ and ‘Incongruent × pokotu’ in Supplementary Tables S2(e) and S3(e)). However, there were three exceptions: (a) Japanese participants showed a significantly longer RT to large animals compared with the RT to small animals in the Incongruent condition, which is statistically shown by the pairwise comparisons (Supplementary Figure S2(a)) and the significant interaction term of Incongruent × Small in the LMEM (Supplementary Table S2(a)). (b) German participants showed generally longer RT to front-vowel acoustic stimuli compared with those of the back vowels, which is evident in the significant contribution of the fixed term ‘Category = Front’ in the LMEM (Supplementary Table S3(b)). (c) German participants showed shorter RT to the acoustic stimuli ‘pokotu’ compared with the other back-vowel items (i.e., ‘tokopu’ and ‘kopotu’), which is indicated by the significant contribution of the fixed term ‘Type = pokotu’ in the LMEM (Supplementary Table S3(e)). These exceptions notwithstanding, the results highlight the fact that all stimuli contributed more or less equally to the differences between the experimental conditions, which indicates that cross-modal associations between visual and acoustic stimuli are not driven by intrinsic properties of individual stimuli and cannot be restricted to only one category. It should also be noted that the points (b) and (c) were reported based on the results of the LMEMs, but they are not supported by pairwise comparisons.

### Effect of Perceptual Features on Cross-Modal Semantic Association

**Figure 6.**
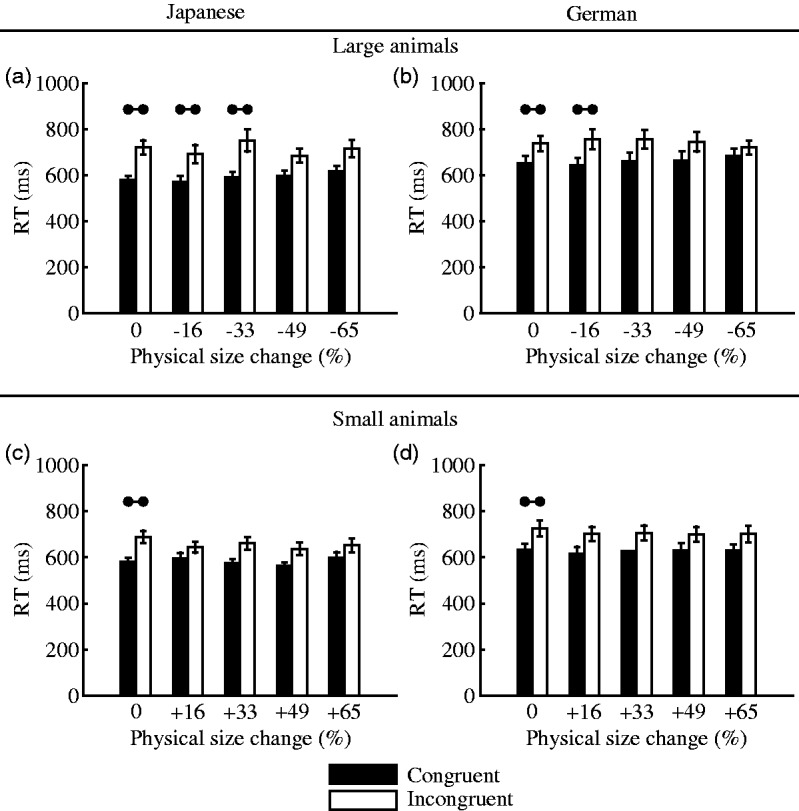
Average RT shown for each experimental condition (Congruent vs. Incongruent) and the physical size of the visual stimuli. The figures were drawn using the dataset for (a) Japanese participants – large animals, (b) German participants – large animals, (c) Japanese participants – small animals, and (d) German participants – small animals. Error bars indicate standard errors. The results of pairwise comparisons are represented by horizontal bars (*p* < .05, Tukey-corrected). RT = response time.

To test our main hypothesis – that is, the effect of perceptual features on semantic associations – we focused on trials with visual stimuli in the experimental blocks alone and studied the effect of physical (screen) size on differences in the RT induced by the experimental conditions (Congruent vs. Incongruent). In other words, we tested whether the differences between the Congruent and Incongruent conditions decreased due to the manipulation of the physical size of the visual stimuli. The results are summarised in Table 3 and Figure 6. According to the LMEM, the fixed covariate of Size was a significant predictor of RT when German participants categorised the large animals (Table 3(b)), which indicates that the RT of the German participants became longer as the physical size of the large animals became smaller in the Congruent condition (because the Congruent condition was used as a reference level), which partly supports our counter hypothesis according to which associations between the visual and acoustic stimuli due to perceptual features can interfere with semantic-level association. In contrast, the interaction term Incongruent × Size was not significant in the same model (Table 3(b)), which indicates that physical size had a comparable effect in both experimental conditions (Congruent and Incongruent) and that the effect was therefore independent of the associations between acoustic and visual stimuli. Furthermore, in the other models (Table 3(a), (c), and (d)), neither the fixed covariate (Size) nor the interaction term (Incongruent × Size) had significant contributions, which again supports the null hypothesis. This finding implies that the physical size of the pictures did not substantially interfere with the association between visual and acoustic stimuli.

**Table 3.** Results of Liner Mixed Effect Model Analyses for RT.

	*B*	*SE*	95% CI		*t*	*p*
(a) Large animals: JP						
Intercept*	574.60	24.03	527.50	621.71	23.92	<.001
Condition = Incongruent*	172.03	29.87	113.47	230.60	5.76	<.001
Size	12.07	7.95	−3.51	27.65	1.52	.129
Trial number	−0.06	0.20	−0.46	0.34	−0.30	.767
Incongruent × Size	−9.90	7.01	−23.76	3.95	−1.41	.161
Incongruent × Trial Number	−0.29	0.28	−0.84	0.26	−1.03	.303
Size × Trial Number	−0.06	0.09	−0.24	0.11	−0.73	.465
(b) Large animals: DE						
Intercept*	640.49	38.84	564.35	716.63	16.49	<.001
Condition = Incongruent*	131.72	35.09	62.92	200.51	3.75	<.001
Size*	15.73	6.99	2.03	29.43	2.25	.024
Trial number	0.05	0.27	−0.49	0.59	0.18	.859
Incongruent × Size	−6.40	6.21	−18.56	5.78	−1.03	.303
Incongruent × Trial Number	−0.50	0.31	−1.10	0.11	−1.61	.107
Size × Trial Number	−0.13	0.08	−0.29	0.02	−1.67	.096
(c) Small animals: JP						
Intercept*	609.99	34.49	542.38	677.59	17.69	<.001
Condition = Incongruent*	150.90	34.65	82.97	218.83	4.35	<.001
Size	−4.28	7.64	−19.26	10.69	−0.56	.575
Trial number	−0.40	0.29	−0.96	0.17	−1.36	.173
Incongruent × Size	−11.88	6.16	−23.96	0.19	−1.93	.054
Incongruent × Trial Number	−0.50	0.29	−1.06	0.07	−1.71	.087
Size × Trial Number	0.07	0.08	−0.09	0.22	0.83	.407
(d) Small animals: DE						
Intercept*	654.11	31.39	592.58	715.64	20.84	<.001
Condition = Incongruent*	129.77	26.66	77.51	182.03	4.87	<.001
Size	−4.52	6.98	−18.20	9.16	−0.65	.517
Trial number	−0.28	0.24	−0.75	0.19	−1.16	.245
Incongruent × Size	−6.15	6.60	−19.09	6.79	−0.93	.352
Incongruent × Trial Number*	−0.43	0.22	−0.85	−0.01	−2.00	.046
Size × Trial Number	0.04	0.08	−0.10	0.19	0.59	.558

*Note*. All models take the RT as dependent variables and include Condition (Training, Congruent, and Incongruent) as a fixed factor and Size and Trial Number as fixed covariates. The model was built using the dataset for (a) Japanese participants – large animals, (b) German participants – large animals, (c) Japanese participants – small animals, and (d) German participants – small animals. JP = Japan; DE = Germany; RT = response time; B = standardised beta coefficient of the predictor; SE = standard error; CI = confidence interval; *t* = *t* value; *p* = level of significance.

*p < .05.

Regarding the results of pairwise comparisons (Figure 6), the RTs between the Congruent and Incongruent conditions were significantly different for visual stimuli presented in the original size (0%) and after minor changes (albeit only in the case of large animals; Japanese: 16% and 33%; German: 16%), whereas they did not differ significantly between the experimental conditions after major modulations of the picture size (i.e., 49% and 65%). Thus, it is possible to speculate that further manipulations of the size might have eventually eliminated the effect of the experimental condition.

**Figure 7.**
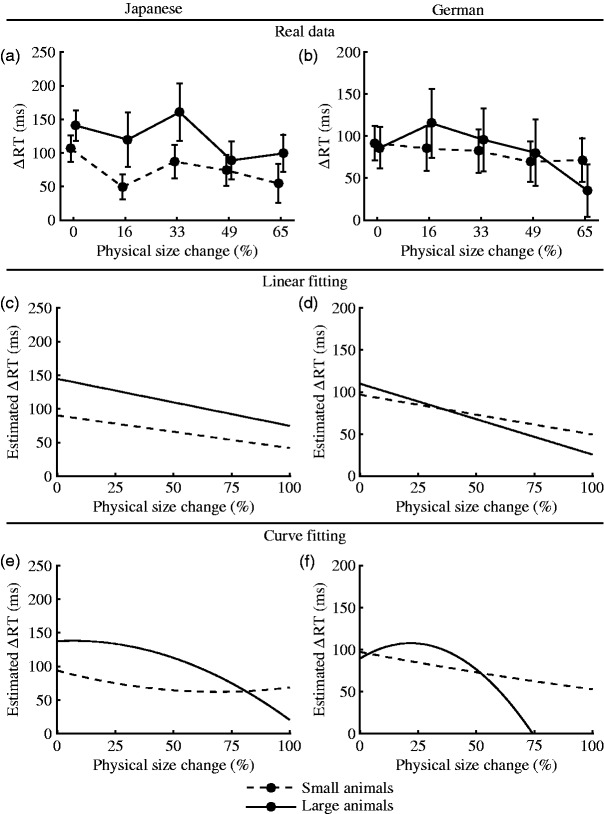
Average ΔRT plotted for each subcategory of visual stimuli (large and small), shown for (a) Japanese and (b) German participants. Estimated ΔRTs by a linear fitting method are shown for (c) Japanese and (d) German participants. Estimated ΔRTs by a curve fitting method are shown for (e) Japanese and (f) German participants. RT = response time.

To investigate the effect in more detail, we plotted the ΔRT (the difference between the averaged RT in the Congruent and Incongruent conditions) against the degree of manipulation of the physical size of the visual stimuli separately for large and small animals (Figure 7(a) and (b)). We fitted the response curves into linear (Figure 7(c) and (d)) and quadratic (Figure 7(e) and (f)) functions and tested whether the estimated linear and quadratic coefficients were significantly different from zero by using a one-sample *t* tests. The visual presentation of the results confirmed that the ΔRT tended to decrease as a function of the level of the manipulation (Figure 7(a) and (b)) for both Japanese and German participants. When fitted into linear and quadratic functions, the ΔRT showed clear decreasing trends as the physical size changed more (Figure 7(c) to (f)). That is, the more the physical size deviated from the original size, the smaller the distance between the averaged RT in the Congruent and Incongruent conditions became. According to the results of the *t* tests, however, neither the linear nor the quadratic component were significantly different from (less than) zero for the Japanese (Table 4(a) and (c)) or the German (Table 4(b) and (d)) data. This finding implies that although there were visible trends indicating that the ΔRT decreased as the physical size changed, the trends were not strong enough to be statistically significant. Thus, we came to the conservative conclusion that the perceptual features of visual stimuli (i.e., physical size) did not substantially influence the association between their semantic features (i.e., their content) or the acoustic stimuli. As we could not observe such an effect on a statistically significant level, our data support the null hypothesis.

**Table 4.** Results of One-Sample *t* Tests for Estimated Linear and Quadratic Coefficients Against Zero.

	*M*	*SE*	95% CI		*t*	*p*
(a) Large animals: JP						
Linear component	−17.96	9.75	−37.78	1.87	−1.84	.074
Quadratic component	−8.93	18.57	−46.66	28.81	−0.48	.634
(b) Large animals: DE						
Linear component	−21.65	11.32	−44.69	1.39	−1.91	.065
Quadratic component	−25.67	14.12	−54.40	3.06	−1.82	.078
(c) Small animals: JP						
Linear component	−12.49	7.12	−26.96	1.98	−1.75	.088
Quadratic component	4.44	11.66	−19.26	28.14	0.38	.706
(d) Small animals: DE						
Linear component	−12.15	9.17	−30.83	6.52	−1.33	.194
Quadratic component	0.54	13.00	−25.93	27.01	0.04	.967

*Note*. JP = Japan; DE = Germany; M = mean; SE = standard error; CI = confidence interval; *t* = *t* value; *p* = level of significance.

## Discussion

Cross-modal or synaesthetic associations are perceived relations between putatively unrelated and nonredundant basic characteristics of sensory input (Spence, 2011). Such cross-modal associations have mostly been studied either in terms of preferences in tasks that ask participants to match stimuli based on introspective considerations or in terms of effects on participants’ performance in implicit tasks, such as speeded classification (Marks, 2004). The fact that cross-modal associations are based on perceptual mapping relates them to language iconicity in the tradition of Peirce, who defined iconicity as a relation between a sign and an object that is based on a ‘mere community in some quality’ (Peirce, 1867, p. 294). It is thus not surprising that there have been attempts to understand sound iconicity (i.e., the association between acoustic qualities of phonemes with nonacoustic qualities) as a specific subcategory of cross-modal association (e.g., Dingemanse et al., 2015; Marks, 1978; Spence, 2011).

It has been shown that humans tend to perceive of qualities of stimuli as being related when they happen to frequently co-occur in nature. Thus, cross-modal associations might be a result of statistical learning in most – if not all – cases (Ernst, 2006, 2007; Parise, Knorre, & Ernst, 2014; Spence, 2011). However, there is evidence suggesting that cross-modal correspondence is not necessarily based on directly perceivable qualities and that it can also be found for complementary (i.e., nonredundant) sensory qualities (Marks, 2013; Parise & Spence, 2013; Spence, 2011, p. 927). In other words, some examples of cross-modal associations, such as those between pitch and brightness (Marks, 1974) or between pitch and taste (Crisinel & Spence, 2009), seem to be arbitrary and not readily explicable through their co-occurrence in nature. A prominent example from the realm of sound iconicity is the seemingly arbitrary association between spiky figures and plosive consonants versus roundish figures and continuants (Fort, Martin, & Peperkamp, 2014; Holland & Wertheimer, 1964; Köhler, 1929; Milán et al., 2013; Nielsen & Rendall, 2013; Ramachandran & Hubbard, 2001). It has thus been suggested that at least some cross-modal associations are triggered by semantic connotations rather than by directly perceivable features (Asano et al., 2015; Hornbostel, 1931; Jürgens & Nikolić, 2012; Karwoski, Odbert, & Osgood, 1942; Lindauer, 1991, 2013; Marks, 1996, 2004; Mroczko-Wąsowicz & Nikolić, 2014; Nikolić, 2009; Walker & Walker, 2016; Walker, Walker, & Francis, 2012).

Hornbostel (1931), for example, proposed the existence of an amodal concept of brightness by which participants can systematically relate visual, olfactory, acoustic, and haptic impressions (see also Börnstein, 1936). Later, Marks revived Hornbostel’s idea, suggesting that cross-modal congruency effects might rely on the discrimination of amodal properties of stimuli (Marks, 1978, 1996, 2004). Similarly, Gallace and Spence (2006) assumed that synaesthetic interactions are not based on purely perceptual processing but involve a semantic categorisation of stimuli. With a slightly different focus but in a similar direction, Karwoski and coworkers studied cross-modal associations between colours and music (Karwoski et al., 1942; Odbert, Karwoski, & Eckerson, 1942). Based on their results, the authors suggested that participants match acoustic and visual impressions based on the emotion or mood they associate with the stimuli. Following this approach, Walker and Walker (2016) suggested a conceptual basis for all cross-modal associations (Walker et al., 2012). That is, the authors assume the existence of a set of abstract features through which sensory impressions of different modalities become comparable, such as brighter, smaller, higher, and so forth (Walker & Walker, 2016).

Thus, cross-modal associations between stimuli of different sensory modalities might be based on a common ground of abstract, amodal concepts. These concepts form continuous, bipolar dimensions by means of which perception can be characterised. Stimuli with adjacent positions on one of these dimensions are perceived as similar (Marks, Hammeal, & Bornstein, 1987). Arguably the most cited dimensional semantic space is the Evaluation–Potency–Activity model introduced by Osgood, Suci, and Tannenbaum (1957), which has alternatively been referred to using the labels *Pleasure*, *Arousal*, and *Dominance* (Bakker, van der Voordt, Vink, & de Boon, 2014; Mehrabian & Russell, 1974; Russell & Mehrabian, 1977). Following this approach, the association of a large object and a low-frequency sound is not necessarily based on actual sensory-perceivable qualities, but on the allocation of the acoustic and the visual impression towards one or the other pole of the Potency/Dominance dimension. Consequently, assuming that cross-modal associations are based on conceptual categorisations implies that such associations are independent of the actual appearance of the stimuli and, in contrast, should rely on their semantic interpretation.

The present study is an attempt to shed light on this issue. To that end, we conducted experiments that were designed in a manner that allowed us to manipulate perceivable features and semantic features independently of each other and thus also to monitor their specific effects on cross-modal associations. For the experiments, we applied a speeded categorisation task (the IAT) to assess the association between vowels’ articulatory–acoustic features and size-related connotations. Based on a long tradition of research on the relation between the phonological characteristics of vowels with the notion of size (for reviews, see Auracher, 2017; Nuckolls, 1999; Schmidtke et al., 2014; Tsur, 2006), we predicted that front vowels should be preferably associated with pictures depicting small animals, whereas back vowels were assumed to be associated with pictures depicting large animals. At the same time, we manipulated the perceivable features of the visual stimuli by changing the physical size of the pictures in a manner that always set perceivable features and semantic features in opposition (e.g., decreasing the size of pictures that displayed large animals).

Our data highlight four major findings: First, we confirmed results of previous studies that have shown that front vowels imply smallness while back vowels are associated with largeness. As the establishment of these cross-modal associations between acoustic and visual stimuli relied on the participants’ ability to infer the relative size of the depicted objects based on their knowledge of the world, our results suggest that the relation between the place of articulation of vowels and the notion of size is based on a semantic interpretation of the stimuli. This finding was also confirmed by the fact that participants’ performance significantly improved over the course of a block in conditions in which nonassociated visual and acoustic stimuli were allocated together, while we found no training effect if the task was consistent with the predicted cross-modal association. To our understanding, the observed training effect indicates that the respective combinations of visual and acoustic stimuli were counterintuitive, thereby requiring participants to practice the task before they were able to automatically categorise the stimuli. Conversely, this means that combinations of visual and acoustic stimuli that showed no training effect were intuitively perceived as being adequate. Our results thereby imply that there was an inherent notion of consistency or inconsistency when matching stimuli across sensory modalities.

Second and most important, we found that directly perceivable features – namely the physical size of the visual stimuli – had only a marginal influence on semantic cross-modal associations. The fact that manipulations of physical size always set perceivable and semantic features in opposition (i.e., the size of the pictures that showed small animals were larger than the size of the pictures that showed large animals, and vice versa) clearly suggests that differences in participants’ performance were dictated by semantic associations, while the influence of perceivable features was insignificant. While these findings corroborate previously reported results, the experimental design applied in this study allowed us to also detect even subtle interactions between perceptual and semantic features. Thus, in contrast to Auracher (2017), our data indicate that manipulations of physical size did exert an effect on associations between sound frequency and picture content. The difference between the response latencies of the two experimental conditions clearly decreased with the degree of manipulation, which suggests a negative correlation. However, the statistical analysis also showed that this effect of manipulations on the perceptual level never outperformed the effect of the semantic association and never reached statistical significance. What is more, while our data showed that the positive effect of semantic associations on participants’ performance decreased to below the level of statistical significance when the physical size of the visual stimuli differed by one third or more from their original size (Figure 6), no significant correlation could be found between the degree of manipulation and the experimentally induced effect on participants’ performance when applying our data to estimate the effect of size manipulation on RT (Figure 7(c) to (f)).

Third, we did not find any indications that our results were confounded by the intrinsic properties of the stimuli used, such as the (phonological characteristic of the) animal names or geometric features of the picture shapes (e.g., curvature, sharpness). Although we did find differences between categories of stimuli, such as between small and large animals for Japanese participants or between front- and back vowels for German participants, it is difficult to interpret these results. We, therefore, assume that these are merely chance effects due to multiple testing. Moreover, the fact that the main effect of the experimental condition on participants’ performance was found for all stimulus Categories and Types clearly suggests that the observed cross-modal associations were due to the conceptual characteristics that distinguished the categories within each modality and not to the specific characteristics of the stimuli used. We also controlled the influence of phonetic congruency by balancing the total occurrence of front vowels and back vowels in the names of the depicted animals. Again, our results do not suggest any measurable influence of phonetic congruency between animal names and acoustic stimuli on participants’ performance.

Finally, our results also suggest that the discovered cross-modal associations do not seem to be dependent on language-specific or cultural-specific factors. As we conducted the same experiment at two different sites (i.e., in Japan and Germany), we could confirm that cultural differences between participants exerted no critical influence on the association between the notion of size and the tested phonetic characteristics or on the dominance of semantic features over perceptual features in establishing this sound-iconic relation. Although we did find intercultural differences (e.g., regarding the longer RT of German participants to acoustic stimuli or the fact that Germans placed more emphasis on accuracy whereas Japanese participants tended to sacrifice accuracy for speed), these differences only affected minor issues that did not confound the aforementioned major findings. In addition, to investigate the effect of linguistic background more closely, we have performed additional analysis with bilingual participants (five for German and two for Japanese participants) excluded. The results of the monolingual dataset did not show any major differences to the overall results (see Supplementary materials, for details), suggesting that the participants’ linguistic background did not have a critical influence on the cross-model association.

A limitation in the present study is that the average accuracy was extremely high (>90%) for participants from both sites. Consequently, the effect of the experimental conditions (i.e., Congruent vs. Incongruent) on the accuracy data was minimised. Although we found that the accuracy was significantly different between the conditions (Figure 4(a) and (b), Table 1(a) and (b)), the pattern was not as clear as were the results we found for RT (Figure 4(c) and (d), Table 1(c) and (d)). We assume that there was a trade-off between accuracy and RT in that a participant’s focus on either of these two measurements was automatically at the expense of the other one. In other words, we believe that participants took great pains to avoid making erratic responses at the cost of longer response latencies. Thus, attempts to reduce the high level of accuracy (e.g., by introducing a time limit) would have automatically had consequences for the effect of the experimental conditions on the response latency.

## Conclusion

Our findings strongly suggest that sound iconicity involves a semantic interpretation of acoustic characteristics of phonemes that triggers their mapping onto nonacoustic qualities. In contrast, directly perceivable features seem to have only a marginal influence on such semantically motivated cross-modal associations. Our results also indicate that these findings are independent of the cultural background of the participants and thus support the claim that such semantic cross-modal associations have a biological rather than a sociocultural basis. We assume that the sound iconicity of magnitude tested here (i.e., the association between articulatory–acoustic features of vowels with the notion of size) is related to the manner by which animals and humans apply sound frequency to signal physical and social hierarchy. This assumption implies that acoustic characteristics can be used in verbal interaction to convey a sense of high or low dominance.

## Supplemental Material

Supplemental material for Semantic Associations Dominate Over Perceptual Associations in Vowel–Size Iconicity Click here for additional data file. Supplemental Material for Semantic Associations Dominate Over Perceptual Associations in Vowel–Size Iconicity by Hideyuki Hoshi Department of Language and Literature, Max Planck Institute for Empirical Aesthetics, Frankfurt, Germany Nahyun Kwon, Kimi Akita and Jan Auracher in i-Perception
