# Supplementary material for: Semantic Associations Dominate Over Perceptual Associations in Vowel–Size Iconicity
Source: Iperception. 2019 Jul 12;10(4):2041669519861981. doi: 10.1177/2041669519861981 (PMC6628535; doi:10.1177/2041669519861981)
Supplement: Supplemental material for Semantic Associations Dominate Over Perceptual Associations in Vowel–Size Iconicity [file Supplemental_Material.pdf]

[Supplementary materials]

Semantic Associations Dominate over Perceptual Associations in Vowel–Size Iconicity

Hideyuki Hoshi

Department of Language and Literature, Max Planck Institute for Empirical Aesthetics, Frankfurt, Germany

Nahyun Kwon

Department of English Linguistics, Graduate School of Humanities, Nagoya University, Nagoya, Japan

Kimi Akita

Department of English Linguistics, Graduate School of Humanities, Nagoya University, Nagoya, Japan

Jan Auracher*

Department of Language and Literature, Max Planck Institute for Empirical Aesthetics, Frankfurt, Germany

Table S1. Information about the visual stimuli used in the present study, showing Area (i.e., the number of the pixels on the screen when the stimulus was presented in original size) and Japanese and German translations with phonetic transcriptions in IPA format and Ratios of front vowels vs. back vowels per word. The front vowels and back vowels are underlined. As the evaluation of phonetic characteristics was based on perceptual similarities between the acoustic stimuli and the articulatory realisation of the words we did not include the open vowel (/a/), shwa (e.g., the final /e/ in German ‘Katze’) and chōon [長音] (e.g., /u/ in Japanese ‘zou’) in the front-vowel / back-vowel ratio as these vowels were not used in the pseudo-words.

**English**		**Japanese**			**German**		
**Name**	**Area (px^2)**	**Name**	**IPA**	**Ratio**	**Name**	**IPA**	**Ratio**
Elephant	1,462,051	Zou	*zo:*	0:1	Elefant	*eləˈfant*	2:0
Hippopotamus	1,462,199	Kaba	*kaba*	0:0	Nilpferd	*niːlˌpfeːɐ̯t*	2:0
Rhinoceros	1,461,482	Sai	*sai*	1:0	Nashorn	naːshoːrn	0:1
Cat	1,462,229	Neko	*neko*	1:1	Katze	*kaʦə*	0:0
Rabbit	1,461,845	Usagi	*ɯsagi*	1:1	Hase	*haːzə*	0:0
Hamster	1,461,149	Hamusta-	*hamɯ̥sɯ̥ta:*	0:1	Hamster	*hamstɐ*	0:0

Note. IPA: International Phonetic Alphabet notation.

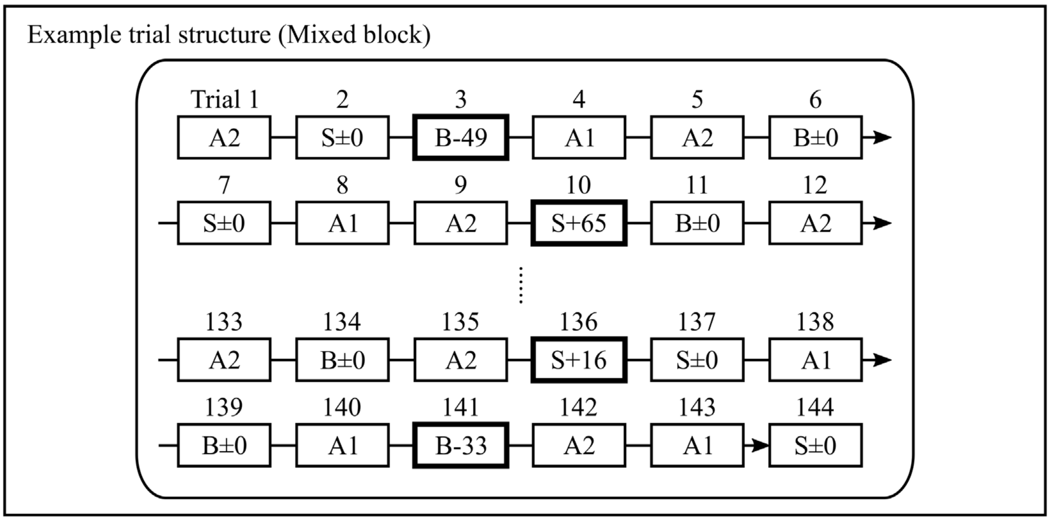

Figure S1. Schematic representation of trial structures in the mixed block (Blocks 3 and 5). A1: back-vowel auditory stimulus; A2: front-vowel auditory stimulus; B-X: large-animal (visual) stimulus presented in 100-X% in physical size; S+X: small-animal (visual) stimulus presented in 100+X% in physical size.

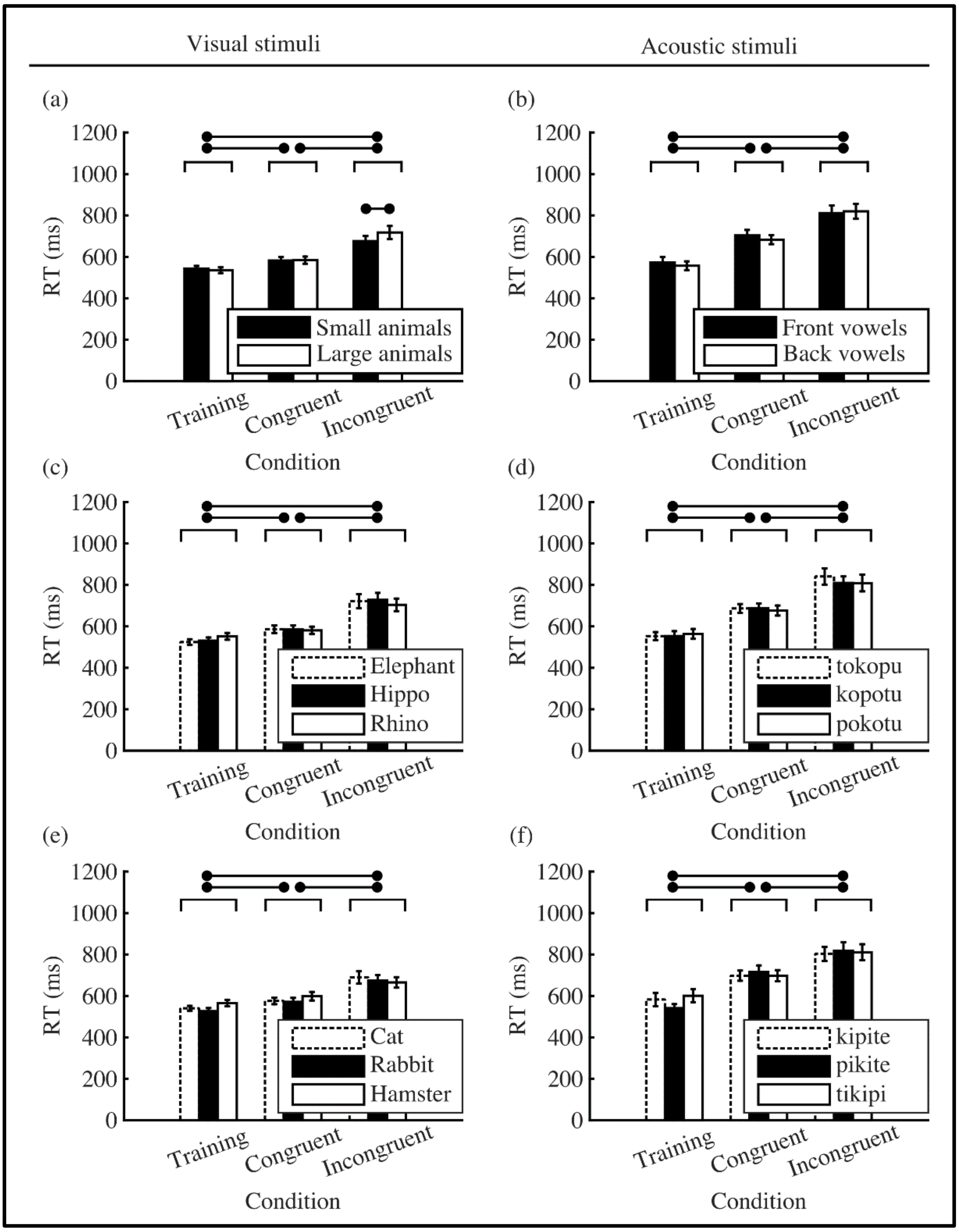

Figure S2. Average RT for Japanese participants, shown for each condition and for each (a) visual category, (b) auditory category, (c) type in large-animal category, (d) type in back-vowel-word category, (e) type in small-animal category, and (f) type in front-vowel-word category. Error bars indicate the Standard Errors. The results of pairwise comparisons are represented by horizontal bars ($p < .05$, Tukey-corrected).

Table S2. The results of LMEM analyses for RT of Japanese participants. All models take RT as dependent variables and include Condition (Training, Congruent, and Incongruent) as a fixed factor (reference level = Congruent condition) and Trial number as a fixed covariate. Each model differs regarding an additionally included fixed factor: (a) Visual category (reference = Large animal), (b) Auditory category (reference = Back vowel), (c) Large-animal type (reference = Elephant), (d) Small-animal type (reference = Cat), (e) Back-vowel-word type (reference = tokopu), and (f) Front-vowel-word type (reference = kipite). The asterisks indicate the terms that contributed significantly to the model (*p* < .05).

(a) Visual categories: JP	*B*	*SE*	95% *CI*		*t*	*p*
* Intercept	592.75	22.46	548.73	636.77	26.40	< .001
Condition = Training	-12.73	24.38	-60.52	35.07	-0.52	0.602
* Condition = Incongruent	161.97	27.24	108.58	215.35	5.95	< .001
Category = Small	12.77	16.27	-19.11	44.66	0.79	0.432
Trial number	-0.12	0.15	-0.42	0.17	-0.82	0.414
Training × Small	1.04	16.90	-32.09	34.16	0.06	0.951
* Incongruent × Small	-38.76	14.26	-66.71	-10.80	-2.72	0.007
* Training × Trial number	-2.32	0.66	-3.62	-1.02	-3.50	< .001
* Incongruent × Trial number	-0.41	0.21	-0.81	< 0.01	-1.98	0.048
Small × Trial number	-0.20	0.19	-0.56	0.17	-1.07	0.284
(b) Auditory categories: JP	*B*	*SE*	95% *CI*		*t*	*p*
* Intercept	683.11	27.23	629.73	736.49	25.09	< .001
* Condition = Training	-53.33	25.89	-104.07	-2.59	-2.06	0.039
* Condition = Incongruent	180.44	28.00	125.57	235.32	6.45	< .001
Category = Front	29.13	17.70	-5.57	63.83	1.65	0.100
Trial number	< 0.01	0.17	-0.35	0.34	-0.02	0.983
Training × Front	-8.90	22.50	-53.01	35.21	-0.40	0.692
Incongruent × Front	-28.30	15.82	-59.30	2.70	-1.79	0.074
* Training × Trial number	-3.95	0.89	-5.69	-2.21	-4.45	< .001
* Incongruent × Trial number	-0.60	0.24	-1.07	-0.14	-2.54	0.011
Front × Trial number	-0.11	0.17	-0.44	0.22	-0.65	0.514
(c) Large animal: JP	*B*	*SE*	95% *CI*		*t*	*p*
* Intercept	598.48	30.80	538.09	658.87	19.43	< .001
Condition = Training	-40.92	32.81	-105.24	23.41	-1.25	0.212
* Condition = Incongruent	155.64	32.22	92.47	218.80	4.83	< .001
Type = Hippopotamus	-3.18	23.19	-48.65	42.29	-0.14	0.891
Type = Rhinoceros	-1.92	23.57	-48.13	44.28	-0.08	0.935
Trial number	-0.18	0.25	-0.67	0.30	-0.75	0.452
Training × Hippopotamus	15.54	27.17	-37.72	68.80	0.57	0.567
Incongruent × Hippopotamus	6.99	25.32	-42.64	56.62	0.28	0.782
Training × Rhinoceros	31.48	28.22	-23.85	86.81	1.12	0.265
Incongruent × Rhinoceros	-10.31	22.37	-54.16	33.55	-0.46	0.645
* Training × Trial number	-1.80	0.84	-3.44	-0.15	-2.14	0.032
Incongruent × Trial number	-0.30	0.28	-0.85	0.25	-1.08	0.282
Hippopotamus × Trial number	0.05	0.24	-0.43	0.53	0.19	0.850
Rhinoceros × Trial number	-0.02	0.26	-0.54	0.50	-0.07	0.941
(d) Small animal: JP	*B*	*SE*	95% *CI*		*t*	*p*
* Intercept	615.86	27.79	561.39	670.33	22.17	< .001
Condition = Training	-6.12	33.02	-70.85	58.60	-0.19	0.853

* Condition = Incongruent	149.85	34.14	82.92	216.79	4.39	< .001
Type = Rabbit	-44.60	23.47	-90.61	1.42	-1.90	0.057
Type = Hamster	3.63	25.87	-47.08	54.35	0.14	0.888
* Trial number	-0.53	0.25	-1.01	-0.04	-2.13	0.034
Training × Rabbit	11.28	27.27	-42.17	64.74	0.41	0.679
Incongruent × Rabbit	-8.17	23.63	-54.50	38.17	-0.35	0.730
Training × Hamster	10.62	29.11	-46.45	67.69	0.36	0.715
Incongruent × Hamster	-44.08	27.56	-98.10	9.95	-1.60	0.110
* Training × Trial number	-2.85	0.91	-4.63	-1.07	-3.14	0.002
Incongruent × Trial number	-0.53	0.31	-1.14	0.08	-1.70	0.089
* Rabbit × Trial number	0.52	0.24	0.04	0.99	2.14	0.032
Hamster × Trial number	0.23	0.28	-0.33	0.78	0.80	0.426
(e) Back-vowel word: JP	*B*	*SE*	95% *CI*		*t*	*p*
* Intercept	691.69	30.72	631.48	751.91	22.52	< .001
* Condition = Training	-84.87	37.33	-158.04	-11.69	-2.27	0.023
* Condition = Incongruent	194.05	36.89	121.73	266.38	5.26	< .001
Type = kopotu	7.72	24.38	-40.07	55.51	0.32	0.752
Type = pokotu	-26.91	26.06	-77.99	24.18	-1.03	0.302
Trial number	-0.06	0.23	-0.51	0.39	-0.26	0.791
Training × kopotu	7.44	36.51	-64.12	79.01	0.20	0.838
Incongruent × kopotu	-27.86	25.19	-77.25	21.52	-1.11	0.269
Training × pokotu	30.70	36.89	-41.62	103.03	0.83	0.405
Incongruent × pokotu	-18.26	24.71	-66.69	30.18	-0.74	0.460
* Training × Trial number	-2.99	1.25	-5.43	-0.54	-2.40	0.017
Incongruent × Trial number	-0.58	0.31	-1.20	0.03	-1.85	0.064
kopotu × Trial number	-0.13	0.26	-0.65	0.39	-0.49	0.627
pokotu × Trial number	0.22	0.30	-0.36	0.80	0.74	0.460
(f) Front-vowel word: JP	*B*	*SE*	95% *CI*		*t*	*p*
* Intercept	691.90	35.39	622.53	761.28	19.55	< .001
Condition = Training	-21.84	37.46	-95.28	51.60	-0.58	0.560
* Condition = Incongruent	153.97	31.49	92.23	215.71	4.89	< .001
Type = pikite	21.89	26.38	-29.83	73.60	0.83	0.407
Type = tikipi	36.42	28.03	-18.52	91.37	1.30	0.194
Trial number	0.09	0.31	-0.52	0.69	0.28	0.780
Training × pikite	-59.47	41.14	-140.12	21.18	-1.45	0.148
Incongruent × pikite	-6.35	25.37	-56.09	43.39	-0.25	0.802
Training × tikipi	-14.57	37.61	-88.30	59.17	-0.39	0.699
Incongruent × tikipi	6.24	23.12	-39.08	51.56	0.27	0.787
* Training × Trial number	-4.76	1.23	-7.17	-2.35	-3.87	< .001
* Incongruent × Trial number	-0.65	0.29	-1.21	-0.09	-2.26	0.024
pikite × Trial number	-0.06	0.29	-0.63	0.52	-0.19	0.846
tikipi × Trial number	-0.50	0.31	-1.11	0.11	-1.61	0.108

Note: JP: Japan; DE: Germany; RT: reaction time; B: standardised beta coefficient of the predictor; SE: standard error; CI: confidence interval; t: t-value; *p*: level of significance; *p < .05.

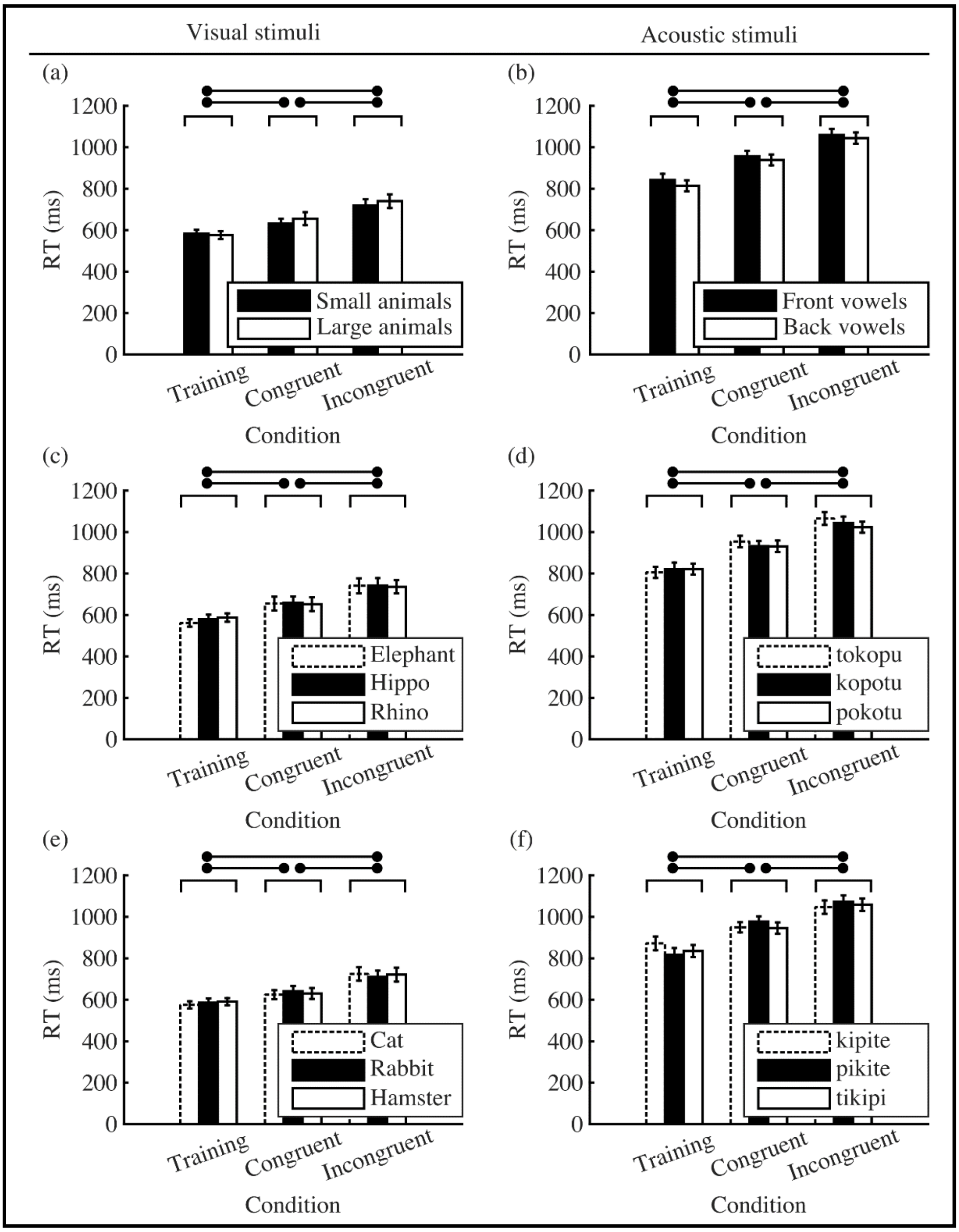

Figure S3. Average RT for German participants, shown for each condition and for each (a) visual category, (b) auditory category, (c) type in large-animal category, (d) type in back-vowel-word category, (e) type in small-animal category, and (e) type in front-vowel-word category. Error bars indicate the *SE*s. The results of pairwise comparisons are represented by horizontal bars ($p < .05$, Tukey-corrected).

Table S3. The results of the LMEM analyses for RT of the German participants. All models take RT as dependent variables and include Condition (Training, Congruent, and Incongruent) as a fixed factor (reference level = Congruent condition) and Trial number as a fixed covariate. Each model differs regarding an additionally included fixed factor: (a) Visual category (reference = Large animal), (b) Auditory category (reference = Back vowel), (c) Large-animal type (reference = Elephant), (d) Small-animal type (reference = Cat), (e) Back-vowel-word type (reference = tokopu), and (f) Front-vowel-word type (reference = kipite). The asterisks indicate the terms that contributed significantly to the model ($p < .05$).

(a) Visual categories: DE	*B*	*SE*	95% *CI*		*t*	*p*
* Intercept	670.72	37.40	597.41	744.02	17.93	< .001
* Condition = Training	-58.13	25.02	-107.17	-9.08	-2.32	0.020
* Condition = Incongruent	116.19	29.54	58.29	174.09	3.93	< .001
Category = Small	-23.21	17.15	-56.82	10.40	-1.35	0.176
Trial number	-0.21	0.23	-0.65	0.23	-0.92	0.356
Training × Small	33.74	18.72	-2.96	70.43	1.80	0.072
Incongruent × Small	3.49	17.74	-31.28	38.26	0.20	0.844
* Training × Trial number	-1.84	0.54	-2.89	-0.79	-3.43	0.001
Incongruent × Trial number	-0.45	0.24	-0.91	0.02	-1.89	0.058
Small × Trial number	-0.01	0.15	-0.31	0.29	-0.05	0.960
(b) Auditory categories: DE	*B*	*SE*	95% *CI*		*t*	*p*
* Intercept	951.17	30.00	892.35	1010.00	31.70	< .001
* Condition = Training	-77.53	24.96	-126.46	-28.60	-3.11	0.002
* Condition = Incongruent	129.19	24.97	80.25	178.14	5.17	< .001
* Category = Front	33.23	16.41	1.07	65.39	2.03	0.043
Trial number	-0.17	0.18	-0.51	0.18	-0.96	0.338
Training × Front	2.27	24.71	-46.17	50.70	0.09	0.927
Incongruent × Front	-4.37	14.71	-33.20	24.47	-0.30	0.767
* Training × Trial number	-3.16	0.98	-5.09	-1.24	-3.22	0.001
Incongruent × Trial number	-0.32	0.20	-0.72	0.07	-1.60	0.109
Front × Trial number	-0.20	0.16	-0.50	0.11	-1.26	0.207
(c) Large-animal type: DE	*B*	*SE*	95% *CI*		*t*	*p*
* Intercept	669.42	42.97	585.18	753.65	15.58	< .001
* Condition = Training	-80.17	31.49	-141.91	-18.43	-2.55	0.011
* Condition = Incongruent	118.62	38.76	42.63	194.61	3.06	0.002
Type = Hippopotamus	0.46	22.83	-44.29	45.20	0.02	0.984
Type = Rhinoceros	4.74	24.15	-42.60	52.09	0.20	0.844
Trial number	-0.19	0.29	-0.76	0.37	-0.68	0.500
Training × Hippopotamus	27.70	28.26	-27.69	83.09	0.98	0.327
Incongruent × Hippopotamus	-1.80	22.76	-46.41	42.82	-0.08	0.937
Training × Rhinoceros	29.28	28.89	-27.36	85.91	1.01	0.311
Incongruent × Rhinoceros	0.14	21.83	-42.66	42.94	0.01	0.995
* Training × Trial number	-1.68	0.82	-3.28	-0.07	-2.05	0.040
Incongruent × Trial number	-0.48	0.31	-1.09	0.14	-1.52	0.129
Hippopotamus × Trial number	0.07	0.26	-0.44	0.58	0.25	0.800
Rhinoceros × Trial number	-0.12	0.29	-0.69	0.46	-0.40	0.686
(d) Small-animal type: DE	*B*	*SE*	95% *CI*		*t*	*p*
* Intercept	656.34	31.94	593.72	718.96	20.55	< .001
Condition = Training	-23.68	27.47	-77.53	30.17	-0.86	0.389
* Condition = Incongruent	131.95	27.39	78.26	185.65	4.82	< .001

	Type = Rabbit	-14.58	22.72	-59.12	29.96	-0.64	0.521
	Type = Hamster	-11.71	22.33	-55.48	32.07	-0.52	0.600
	Trial number	-0.44	0.22	-0.88	< 0.01	-1.95	0.051
	Training × Rabbit	4.76	25.57	-45.36	54.88	0.19	0.852
	Incongruent × Rabbit	-33.16	20.50	-73.34	7.02	-1.62	0.106
	Training × Hamster	13.48	26.05	-37.57	64.54	0.52	0.605
	Incongruent × Hamster	-10.23	19.33	-48.12	27.66	-0.53	0.597
*	Training × Trial number	-2.20	0.72	-3.62	-0.78	-3.04	0.002
	Incongruent × Trial number	-0.43	0.22	-0.87	< 0.01	-1.95	0.051
	Rabbit × Trial number	0.44	0.25	-0.04	0.93	1.79	0.074
	Hamster × Trial number	0.26	0.25	-0.23	0.74	1.04	0.297
	(e) Back-vowel-word type: DE	*B*	*SE*	95% *CI*		*t*	*p*
*	Intercept	981.60	36.36	910.31	1052.90	27.00	< .001
*	Condition = Training	-126.26	38.14	-201.03	-51.49	-3.31	0.001
*	Condition = Incongruent	136.15	32.52	72.39	199.91	4.19	< .001
	Type = kopotu	-20.93	29.03	-77.84	35.99	-0.72	0.471
*	Type = pokotu	-68.35	28.98	-125.16	-11.54	-2.36	0.018
	Trial number	-0.39	0.27	-0.92	0.13	-1.47	0.141
	Training × kopotu	60.20	39.24	-16.74	137.13	1.53	0.125
	Incongruent × kopotu	-2.48	24.68	-50.86	45.91	-0.10	0.920
	Training × pokotu	70.57	39.38	-6.63	147.78	1.79	0.073
	Incongruent × pokotu	-21.85	23.60	-68.11	24.41	-0.93	0.355
*	Training × Trial number	-2.85	1.43	-5.66	-0.05	-2.00	0.046
	Incongruent × Trial number	-0.31	0.24	-0.78	0.16	-1.31	0.190
	kopotu × Trial number	-0.01	0.30	-0.59	0.57	-0.04	0.969
*	pokotu × Trial number	0.64	0.32	0.02	1.26	2.02	0.043
	(f) Front-vowel-word type: DE	*B*	*SE*	95% *CI*		*t*	*p*
*	Intercept	981.22	31.93	918.62	1043.80	30.73	< .001
	Condition = Training	-29.74	46.31	-120.52	61.05	-0.64	0.521
*	Condition = Incongruent	119.86	31.57	57.98	181.75	3.80	< .001
	Type = pikite	23.92	29.78	-34.46	82.29	0.80	0.422
	Type = tikipi	-21.13	24.72	-69.59	27.34	-0.85	0.393
	Trial number	-0.41	0.26	-0.92	0.10	-1.59	0.113
	Training × pikite	-64.49	38.47	-139.91	10.92	-1.68	0.094
	Incongruent × pikite	-3.54	22.83	-48.30	41.22	-0.15	0.877
	Training × tikipi	-21.36	36.25	-92.43	49.71	-0.59	0.556
	Incongruent × tikipi	13.47	25.30	-36.13	63.07	0.53	0.595
*	Training × Trial number	-3.97	1.28	-6.48	-1.46	-3.10	0.002
	Incongruent × Trial number	-0.31	0.27	-0.84	0.21	-1.16	0.246
	pikite × Trial number	0.02	0.35	-0.66	0.70	0.07	0.944
	tikipi × Trial number	0.21	0.28	-0.33	0.75	0.75	0.454

Note: JP: Japan; DE: Germany; RT: reaction time; B: standardised beta coefficient of the predictor; SE: standard error; CI: confidence interval; t: t-value; p: level of significance; *p < .05.

Table r1. (Monolingual data only) The results of the LMEM analyses performed on accuracy (a and b) and the RT (c and d) data. (a) Japanese monolingual participants – accuracy, (b) German monolingual participants – accuracy, (c) Japanese monolingual participants – RT, and (d) German monolingual participants – RT. Congruent condition and Visual modality are used as a reference level in the analysis.

(a) Accuracy: JP	*B*	*SE*	95% *CI*		*t*	*p*
* Intercept	94.61	0.96	92.72	96.51	98.39	< .001
* Condition = Training	1.73	0.74	0.26	3.19	2.32	0.021
* Condition = Incongruent	-3.47	1.54	-6.51	-0.43	-2.25	0.025
* Modality = Auditory	1.41	0.59	0.25	2.57	2.40	0.017
Training × Auditory	0.23	0.78	-1.31	1.77	0.30	0.767
Incongruent × Auditory	0.36	1.52	-2.64	3.36	0.24	0.814
(b) Accuracy: DE	*B*	*SE*	95% *CI*		*t*	*p*
* Intercept	95.95	1.41	93.18	98.73	68.22	< .001
Condition = Training	1.60	1.39	-1.14	4.35	1.15	0.251
Condition = Incongruent	0.26	1.36	-2.42	2.95	0.19	0.847
* Modality = Auditory	0.93	0.36	0.23	1.64	2.61	0.010
Training × Auditory	-0.41	1.22	-2.82	2.00	-0.33	0.739
Incongruent × Auditory	-2.35	1.35	-5.01	0.32	-1.74	0.084
(c) RT: JP	*B*	*SE*	95% *CI*		*t*	*p*
* Intercept	589.85	21.09	548.52	631.19	27.97	< .001
Condition = Training	10.85	20.19	-28.71	50.42	0.54	0.591
* Condition = Incongruent	156.18	23.85	109.44	202.92	6.55	< .001
* Modality = Auditory	111.72	17.25	77.90	145.54	6.48	< .001
Trial number	-0.14	0.13	-0.40	0.12	-1.03	0.301
* Training × Auditory	-86.93	19.56	-125.26	-48.60	-4.44	< .001
Incongruent × Auditory	6.23	15.82	-24.79	37.25	0.39	0.694
* Training × Trial number	-2.95	0.56	-4.05	-1.86	-5.29	< .001
* Incongruent × Trial number	-0.56	0.18	-0.91	-0.20	-3.10	0.002
Auditory × Trial number	0.07	0.14	-0.21	0.34	0.47	0.637
(d) RT: DE	*B*	*SE*	95% *CI*		*t*	*p*
* Intercept	665.99	35.04	597.31	734.67	19.01	< .001
Condition = Training	-35.85	20.79	-76.61	4.90	-1.72	0.085
* Condition = Incongruent	109.87	26.67	57.58	162.15	4.12	< .001
* Modality = Auditory	305.65	18.39	269.60	341.70	16.62	< .001
Trial number	-0.35	0.21	-0.76	0.07	-1.65	0.099
* Training × Auditory	-63.27	24.37	-111.03	-15.51	-2.60	0.009
Incongruent × Auditory	21.73	18.83	-15.18	58.64	1.15	0.249
* Training × Trial number	-2.28	0.50	-3.27	-1.30	-4.56	< .001
Incongruent × Trial number	-0.42	0.22	-0.85	0.01	-1.91	0.057
Auditory × Trial number	0.03	0.13	-0.23	0.29	0.23	0.815

Note: JP: Japan; DE: Germany; RT: reaction time; B: standardised beta coefficient of the predictor; SE: standard error; CI: confidence interval; t: t-value; p: level of significance; *p < .05.

Table r2. (Monolingual data only) Results of one-sample *t*-tests for estimated linear coefficients of training curve compared against zero.

(a) JP	*M*	*SE*	95% *CI*		*t*	*p*
* Training: Visual	-27.21	6.42	-40.28	-14.14	-4.24	< .001
* Training: Auditory	-42.00	7.81	-57.91	-26.10	-5.38	< .001
Congruent: Visual	-7.54	5.23	-18.19	3.11	-1.44	0.159
Congruent: Auditory	-1.63	7.17	-16.23	12.96	-0.23	0.821
* Incongruent: Visual	-27.77	7.60	-43.25	-12.29	-3.65	0.001
* Incongruent: Auditory	-27.12	10.46	-48.43	-5.80	-2.59	0.014
(b) DE	*M*	*SE*	95% *CI*		*t*	*p*
* Training: Visual	-19.78	4.74	-29.48	-10.08	-4.18	< .001
* Training: Auditory	-42.24	10.59	-63.93	-20.54	-3.99	< .001
Congruent: Visual	-14.05	9.13	-32.75	4.65	-1.54	0.135
Congruent: Auditory	-13.19	7.10	-27.74	1.36	-1.86	0.074
* Incongruent: Visual	-33.42	10.03	-53.96	-12.87	-3.33	0.002
* Incongruent: Auditory	-29.59	9.13	-48.30	-10.89	-3.24	0.003

Note: JP: Japan; DE: Germany; M: mean; SE: standard error; CI: confidence interval; t: t-value; p: level of significance; *p < .05.

Table r3. (Monolingual data only) The results of LMEM analyses for RT. All models take the RT as dependent variables and include Condition (Training, Congruent, and Incongruent) as a fixed factor and Size and Trial number as fixed covariates. The model was built using the dataset for (a) Japanese monolingual participants – large animals, (b) German monolingual participants – large animals, (c) Japanese monolingual participants – small animals, and (d) German monolingual participants – small animals.

(a) Large animals: JP	*B*	*SE*	95% *CI*		*t*	*p*
* Intercept	573.11	23.86	526.33	619.89	24.02	< .001
* Condition = Incongruent	174.96	31.03	114.13	235.78	5.64	< .001
Size	14.84	7.93	-0.71	30.39	1.87	0.061
Trial number	-0.05	0.21	-0.46	0.36	-0.24	0.808
Incongruent × Size	-11.71	7.19	-25.81	2.38	-1.63	0.103
Incongruent × Trial number	-0.24	0.30	-0.82	0.34	-0.81	0.416
Size × Trial number	-0.11	0.09	-0.27	0.06	-1.24	0.216
(b) Large animals: DE	*B*	*SE*	95% *CI*		*t*	*p*
* Intercept	646.08	41.24	565.23	726.94	15.67	< .001
* Condition = Incongruent	124.21	40.36	45.08	203.33	3.08	0.002
* Size	15.78	7.68	0.73	30.83	2.06	0.040
Trial number	-0.06	0.31	-0.66	0.55	-0.19	0.852
Incongruent × Size	-4.67	6.62	-17.64	8.30	-0.71	0.481
Incongruent × Trial number	-0.51	0.35	-1.20	0.18	-1.46	0.146
Size × Trial number	-0.14	0.09	-0.32	0.03	-1.62	0.106
(c) Small animals: JP	*B*	*SE*	95% *CI*		*t*	*p*
* Intercept	586.58	22.82	541.84	631.32	25.70	< .001
* Condition = Incongruent	171.22	33.19	106.15	236.30	5.16	< .001
Size	-0.29	6.95	-13.92	13.34	-0.04	0.966

Trial number	-0.18	0.21	-0.59	0.22	-0.89	0.374
* Incongruent × Size	-13.29	6.11	-25.27	-1.32	-2.18	0.030
* Incongruent × Trial number	-0.66	0.27	-1.20	-0.12	-2.41	0.016
Size × Trial number	0.04	0.08	-0.11	0.19	0.51	0.613
(d) Small animals: DE	*B*	*SE*	95% *CI*		*t*	*p*
* Intercept	663.03	35.57	593.29	732.77	18.64	< .001
* Condition = Incongruent	118.20	28.51	62.31	174.10	4.15	< .001
Size	-5.97	7.40	-20.49	8.54	-0.81	0.420
Trial number	-0.36	0.27	-0.88	0.16	-1.35	0.176
Incongruent × Size	-1.41	6.40	-13.96	11.14	-0.22	0.826
Incongruent × Trial number	-0.47	0.24	-0.94	0.00	-1.95	0.051
Size × Trial number	0.03	0.08	-0.13	0.18	0.32	0.751

Note: JP: Japan; DE: Germany; RT: reaction time; B: standardised beta coefficient of the predictor; SE: standard error; CI: confidence interval; t: t-value; p: level of significance; *$p < .05$.

Table r4. (Monolingual data only) Results of one-sample *t*-tests for estimated linear and quadratic coefficients against zero.

(a) Large animals: JP	*M*	*SE*	95% *CI*		*t*	*p*
Linear component	-18.95	10.28	-39.88	1.98	-1.84	0.074
Quadratic component	-6.65	19.43	-46.23	32.92	-0.34	0.734
(b) Large animals: DE	*M*	*SE*	95% *CI*		*t*	*p*
Linear component	-17.03	9.94	-37.38	3.33	-1.71	0.098
Quadratic component	-28.28	16.31	-61.69	5.14	-1.73	0.094
(c) Small animals: JP	*M*	*SE*	95% *CI*		*t*	*p*
Linear component	-14.41	7.36	-29.40	0.58	-1.96	0.059
Quadratic component	5.04	12.36	-20.13	30.22	0.41	0.686
(d) Small animals: DE	*M*	*SE*	95% *CI*		*t*	*p*
Linear component	-6.07	7.54	-21.81	9.67	-0.79	0.436
Quadratic component	-5.82	14.38	-35.85	24.21	-0.40	0.694

Note. JP: Japan; DE: Germany; M: mean; SE: standard error; CI: confidence interval; t: t-value; p: level of significance.
